# Canopy spectral reflectance indices correlate with yield traits variability in bread wheat genotypes under drought stress

**DOI:** 10.7717/peerj.14421

**Published:** 2022-11-25

**Authors:** Mohammed Mohi-Ud-Din, Md. Alamgir Hossain, Md. Motiar Rohman, Md. Nesar Uddin, Md. Sabibul Haque, Jalal Uddin Ahmed, Hasan Muhammad Abdullah, Mohammad Anwar Hossain, Mohammad Pessarakli

**Affiliations:** 1 Department of Crop Botany, Bangladesh Agricultural University, Mymensingh, Bangladesh; 2 Department of Crop Botany, Bangabandhu Sheikh Mujibur Rahman Agricultural University, Gazipur, Bangladesh; 3 Plant Breeding Division, Bangladesh Agricultural Research Institute, Gazipur, Bangladesh; 4 Department of Agroforestry and Environment, Bangabandhu Sheikh Mujibur Rahman Agricultural University, Gazipur, Bangladesh; 5 Genetics and Plant Breeding, Bangladesh Agricultural University, Mymensingh, Bangladesh; 6 School of Plant Sciences, The University of Arizona, Tucson, Arizona, USA

**Keywords:** Multispectral vegetation indices, Stay green, Canopy temperature depression, Phenotyping, Multivariate analyses

## Abstract

Drought stress is a major issue impacting wheat growth and yield worldwide, and it is getting worse as the world’s climate changes. Thus, selection for drought-adaptive traits and drought-tolerant genotypes are essential components in wheat breeding programs. The goal of this study was to explore how spectral reflectance indices (SRIs) and yield traits in wheat genotypes changed in irrigated and water-limited environments. In two wheat-growing seasons, we evaluated 56 preselected wheat genotypes for SRIs, stay green (SG), canopy temperature depression (CTD), biological yield (BY), grain yield (GY), and yield contributing traits under control and drought stress, and the SRIs and yield traits exhibited higher heritability (H^2^) across the growing years. Diverse SRIs associated with SG, pigment content, hydration status, and aboveground biomass demonstrated a consistent response to drought and a strong association with GY. Under drought stress, GY had stronger phenotypic correlations with SG, CTD, and yield components than in control conditions. Three primary clusters emerged from the hierarchical cluster analysis, with cluster I (15 genotypes) showing minimal changes in SRIs and yield traits, indicating a relatively higher level of drought tolerance than clusters II (26 genotypes) and III (15 genotypes). The genotypes were appropriately assigned to distinct clusters, and linear discriminant analysis (LDA) demonstrated that the clusters differed significantly. It was found that the top five components explained 73% of the variation in traits in the principal component analysis, and that vegetation and water-based indices, as well as yield traits, were the most important factors in explaining genotypic drought tolerance variation. Based on the current study’s findings, it can be concluded that proximal canopy reflectance sensing could be used to screen wheat genotypes for drought tolerance in water-starved environments.

## Introduction

Bread wheat (*Triticum aestivum* L.) is the second most cereal crop widely grown worldwide and is cultivated extensively in Bangladesh (BBS, 2019; FAO, 2021). A variety of environmental factors impact wheat yields; one of the most critical of them is drought. In the 21st century, it is predicted that many regions of the world will experience more frequent, prolonged, and severe droughts as well as more unpredictable rainfall due to global climate change (MoEF, 2009; Schwalm et al., 2017; Trenberth et al., 2014). This foreseen increase in drought episodes will force the soil to become drier, limiting irrigation water and thus constraining wheat production and yield in the future. Therefore, strategies to improve wheat production must be urgently addressed since the main crop yield could be reduced by more than 50% under limited irrigation water (El-Hendawy et al., 2015). Although there are numerous ways to increase wheat production under water-stressed situations, the most feasible alternative for tackling this challenge is to develop novel wheat genotypes having higher yield potential and greater tolerance to drought (Sinclair, 2011). The main hurdle to accelerating the selection of drought-tolerant wheat genotypes is the lack of efficient phenotyping techniques for drought-related traits in breeding programs (Barakat et al., 2016; Elazab et al., 2015). Most of the tools currently used for drought tolerance phenotyping are destructive, costly, laborious, and resource-intensive, especially when evaluating many entries (El-Hendawy et al., 2015). Drought-tolerant wheat genotypes are selected based on their photosynthetic and transpiration efficiency, water availability and retention, biomass buildup, photoassimilate translocation to growing grains, and yield stability (Reynolds, Pask & Mullan, 2012).

Canopy reflectance sensing is a vital high-throughput phenotyping technique for quickly and non-destructively monitoring drought tolerance features across a large number of genotypes (El-Hendawy et al., 2015; Gizaw, Garland-Campbell & Carter, 2016; Sun et al., 2019; Zhou et al., 2022). The canopy reflects a specific wavelength of light due to its physical properties and diverse biochemical and physiological activities. This wavelength is often used to measure the pigment concentration in the canopy, the senescence pattern, photosynthetic activity, biomass accumulation, leaf area, the yield of grains, and plant hydration status (El-Hendawy et al., 2015; Gizaw, Garland-Campbell & Carter, 2016; Prasad et al., 2009, 2007; Sun et al., 2019). Ground-based proximate sensing is a simple, rapid, practical, and cost-effective method for determining the spectral reflectance of the canopy and has been effectively utilized to evaluate a variety of phenotyping features associated with drought resistance (El-Hendawy et al., 2015; Gizaw, Garland-Campbell & Carter, 2016; Sun et al., 2018; Zhou et al., 2022).

The visible (VIS) and near-infrared (NIR) wavelength ranges (λ = 400–700 and >700 nm, respectively) are used to calculate spectral reflectance indices (SRIs). SRIs have been created to forecast a variety of agronomic and physiological parameters. Combining VIS and NIR spectra yields the simple ratio (SR), normalized difference vegetation index (NDVI), green normalized difference vegetation index (GNDVI), plant nitrogen spectral index (PNSI), and enhanced vegetation index (EVI), which are used to detect subtle changes in vegetative greenness, senescence rate, and stay green duration (Babar et al., 2006; Gizaw, Garland-Campbell & Carter, 2016; Liu et al., 2015; Rouse et al., 1973). To get soil adjusted vegetation health status, the modified soil adjusted vegetation index (MSAVI) and optimized soil adjusted vegetation index (OSAVI) are obtained using VIS and NIR wavelengths (Chen, 1996; Rondeaux, Steven & Baret, 1996). As the proxy for anthocyanin and carotenoid composition, the anthocyanin reflectance index (ARI) and modified carotenoid reflectance index (mCRI), respectively, are generated from the spectra of both the VIS and NIR ranges (Gitelson, Keydan & Merzlyak, 2006). The structure-insensitive pigment index (SIPI) and the plant senescence reflectance index (PSRI) are used to determine the carotenoid to chlorophyll *a* ratio and the advancement of leaf senescence, respectively, in the visible and near-infrared bands (Penuelas, Baret & Filella, 1995; Ren, Chen & An, 2017). The photochemical reflectance index (PRI), normalized chlorophyll-pigment ratio index (NCPI), and xanthophyll pigment epoxidation state (XES) are all calculated using reflectance in the visible spectrum of light to determine the abundance and composition of pigments, whereas the normalized water index (NWI) is calculated using reflectance in the near-infrared band to determine the hydration status of plants (Babar et al., 2006; Gizaw, Garland-Campbell & Carter, 2016).

Several researchers have indicated that monitoring SRIs at different stages of plant growth can be an effective technique to estimate grain yield quickly and non-destructively under varied environmental conditions (El-Hendawy et al., 2015; Elsayed, Rischbeck & Schmidhalter, 2015; Gizaw, Garland-Campbell & Carter, 2016; Lobos et al., 2014; Sun et al., 2019; Zhou et al., 2022). However, the combination of remotely sensed SRIs and other yield features is critical in a target environment, since SRIs are highly linked with grain production, biomass accumulation, and leaf area index under dry conditions compared to irrigated conditions (Aparicio et al., 2000; Gizaw, Garland-Campbell & Carter, 2016).

Boosting the yield potential of a crop in water-limited environments is the most intimidating issue breeders face (Pouri et al., 2019). Grain yield is frequently used as the foundation for selection in drought tolerance breeding, although it is a multifaceted, late-stage phenotype that is influenced by numerous variables other than drought (Khadka et al., 2020). In the breeding program for drought-tolerant wheat varieties, phenotypic-based selection is a conventional practice. However, researchers have discovered several other tools for the selection of wheat genotypes, such as proximal canopy reflectance sensing, to boost breeding efficiency even more (Khadka et al., 2020). During the dry winter months (November to April), most of Bangladesh’s wheat crop is farmed without irrigation (Hossain & Teixeira da Silva, 2013). In Bangladesh, most of the selection approaches for drought-tolerant wheat genotypes were based on the conventional and labor-intensive evaluation of morpho-phenological characters and yield stability (Hannan et al., 2021; Rahman et al., 2016).

Therefore, canopy reflectance sensing could be an effective alternative tool for phenotyping drought-related traits because it can be used to differentiate a large pool of genotypes rapidly and economically. Additionally, understanding the relationships between crop canopy’s SRIs and crop growth and yield traits will aid in identifying drought-related traits that act synergistically on grain yield and in devising a selection strategy for drought-tolerant genotypes. Drought tolerance responses in plants can be quite ubiquitous and polygenic in character (Khalili, Pour-Aboughadareh & Naghavi, 2016). Genetic variability is vital in a breeding program for the selection of desirable traits associated with drought tolerance (Azimi, Jozghasemi & Barba-Gonzalez, 2018). Multivariate analysis is the most widely used statistical technique for estimating genetic diversity among genotype sets (Malik et al., 2014), as it provides more realistic, meaningful, and accurate inferences in exploring relationships, classification, and parameter prediction (Böhm, Smidt & Tintner, 2013). Multivariate tools such as cluster, principal component, and linear discriminant analysis were used to assess the genetic diversity in drought tolerance in the genotypes included in the study.

The main goal of this study was to reveal several SRIs that were linked to grain yield both under irrigated and drought conditions in 56 bread wheat genotypes to speed up the selection process for variety development. Specific objectives were outlined to (i) investigate phenotypic associations of canopy SRIs with yield traits under irrigated and drought-stressed environments; and (ii) employ multivariate analysis that combines SRIs and yield traits to identify suitable drought-tolerant wheat genotypes.

## Materials and Methods

The experiment was accompanied with 56 diverse wheat genotypes chosen from a prior study of drought stress tolerance at the seedling stage (Mohi-Ud-Din et al., 2021). Out of them, 17 varieties and one advanced line were collected from Bangladesh Wheat and Maize Research Institute (Dinajpur, Bangladesh) and Bangladesh Institute of Nuclear Agriculture (Mymensingh, Bangladesh), two mutant lines were from ACI Seed, and 36 wheat accessions were from Bangladesh Agricultural Research Institute’s Plant Genetic Resource Center (Gazipur, Bangladesh). Table S1 has further information about the genotypes.

### Location, soil, and climatic conditions

**Figure 1:**
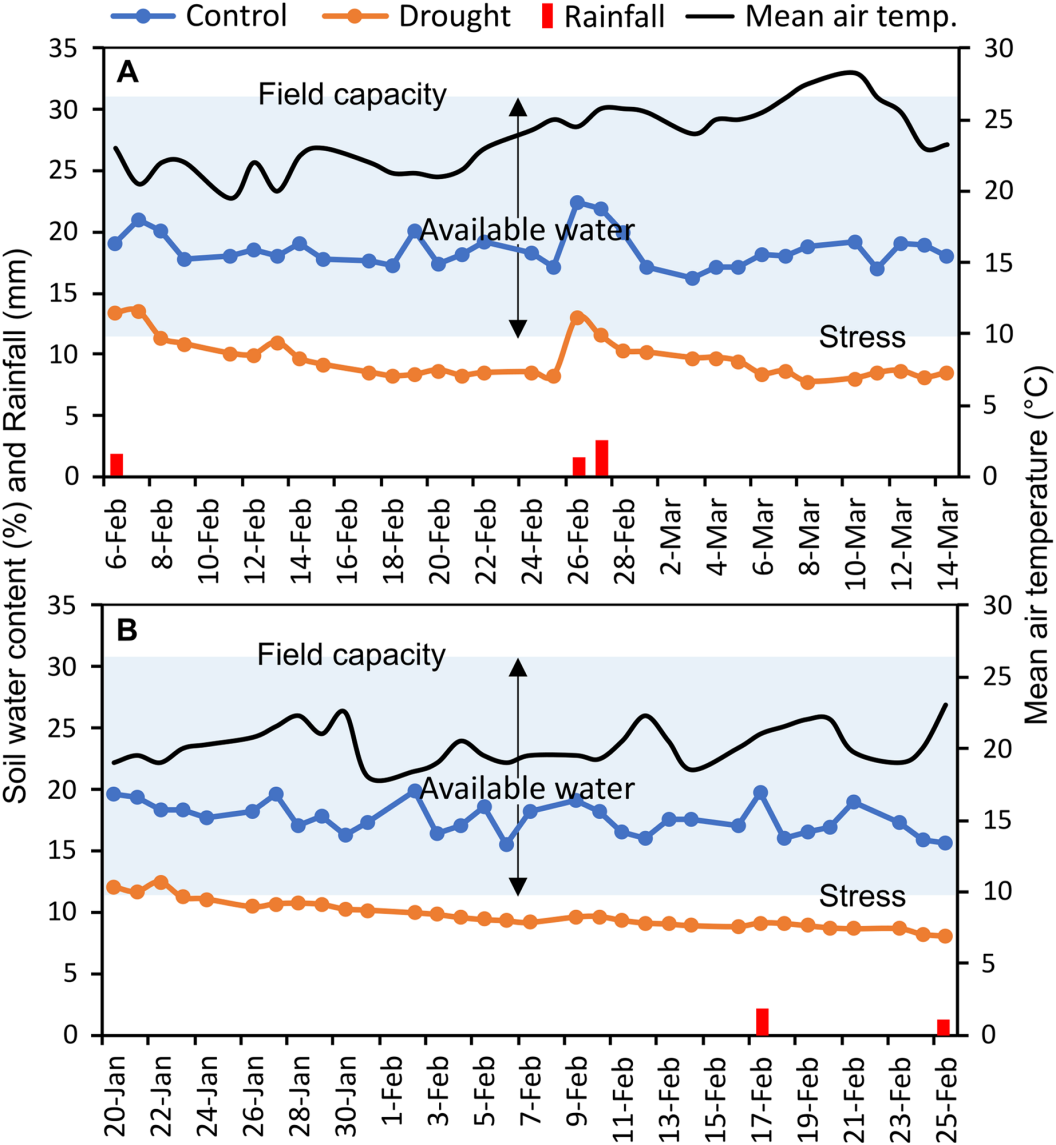
Soil moisture content (%) in each of control and drought-simulated plots, rainfall, and mean air temperature at the reproductive stages of wheat genotypes for two wheat growing seasons. The soil type at the experimental location is silt loam (clay, silt, and sand contributed 24%, 50%, and 26%, respectively), which has a full field capacity at a volumetric soil water content of 30.6 percent. Arrows indicate upper and lower limits of plant available water. Soil water content (%) was measured every day from the randomly selected plots (*n* = 15) of control and drought-treated blocks.

The experiments were carried out at the experimental field of Bangabandhu Sheikh Mujibur Rahman Agricultural University (24.038°N latitude, 90.397°E longitude), Gazipur, Bangladesh, in two consecutive wheat growing seasons (November to April; 2017–18 and 2018–19) (Fig. S1). This location has a typical subtropical monsoon climate. In the study area, bread wheat is typically sown between mid-November and mid-December. The texture of the field soil was silt loam with sand 26%, silt 50%, and clay 24%; and 30.6% volumetric soil water content was required to reach full soil field capacity. The measured soil water content (%) of control and irrigation-limited trials and mean air temperature throughout the reproductive stages were presented in Fig. 1. Monthly weather data of the two wheat growing seasons as well as of the past 10 years (2010–2019) averaged data at the experimental site were summarized in Table S2, and the daily rainfall pattern was presented in Fig. S2.

### Experimental design and treatments

All wheat genotypes were cultivated in two trials maintaining two different moisture regimes: normal irrigated (referred to as “control”) and restricted irrigated (discontinued irrigation after 45 days of seed sowing and termed as “drought”). The experiments were repeated for two consecutive years (2017–’18 and 2018–’19). A randomized complete block design with three replications was employed in all trials.

### Seed sowing and agronomic management

Seeds of all wheat genotypes were sown on 5 December and 18 November during the first (2017–’18) and second (2018–’19) growing years, respectively. Before sowing, seeds were treated with a commercial fungicide containing carboxin and thiram to reduce seedling infection and improve germination and then sown in 20-cm-apart rows at the rate of 12g m^−2^ in the 1.5 m × 1 m unit plots, maintaining a target plant density of approximately 325 seedlings m^−2^. The N, P, K, S, Zn, and B fertilizer dosage of 120−25−90−15−2.5−1 kg ha^−1^ was applied in the commercial form of urea, triple superphosphate (TSP), muriate of potash (MoP), gypsum, zinc sulphate, and boric acid, respectively (Ahmmed et al., 2018). Two-thirds of the nitrogen fertilizer was given as a basal dose in conjunction with the rest of the fertilizers at final land preparation. After the first irrigation (20 days after sowing (DAS)), the remaining ⅓ urea was used as a top dress. During the whole cropping season, five flood irrigations were applied at 20, 45, 60, 70, and 80 DAS in control plots, while only the first two irrigations (20 and 45 DAS) were applied to drought treated plots to allow vegetative growth and then the irrigation was stopped. Approximately 4.35 cm of water was applied to each plot at every irrigation with a mean flow rate of 0.0652 m^3^ min^−1^. Plots were kept weed-free to avoid interference during reflectance measurement. A fungicide containing Azoxystrobin and Difenoconazole was applied at a 10-day interval after 25 days after seedling emergence following label instructions to reduce the perplexing effects of stripe and leaf rust.

### Crop phenology and soil water content

Canopy reflectance, canopy temperature, and leaf greenness (SPAD readings) were collected at heading (GS55), anthesis (GS65), and after 7 (GS71), 14 (GS75), and 21 (GS85) days of anthesis (DAA) (Zadoks, Chang & Konzak, 1974). Heading and anthesis of the genotypes were determined as the visibility, and flowering of 50% of the spikes in a plot, respectively. Since the phenological events differed across genotypes, a schedule was created at the time of anthesis for each genotype for the succeeding measurements of 7, 14, and 21 DAA. Soil water status (%) was recorded daily in randomly 15 selected plots at a depth of 15 cm in each of the control and drought-treated blocks, using a microprocessor-equipped soil water status monitoring device (The PMS-714 from Lutron Electronic Enterprise Co., Ltd. in Taiwan). Averaged daily soil water data were presented in Fig. 1. Using the method of Ogbaga, Stepien & Johnson (2014), the field capacity of the experimental soil was measured at the beginning of the experiment.

### Canopy temperature depression measurement

The canopy temperature was measured according to the procedure of Mohi-Ud-Din et al. (2022). Briefly, a portable infrared thermometer (Model: IR-818, URCERI, USA; the distance-spot ratio of 13:1) was used to record the canopy and ambient air temperature between 11.30 a.m. and 12.30 p.m. (Fig. S3). It took about 30° angle to get to the spotted canopy, which was about 1 m away from the horizontal line. From each plot, a total of 10 records were captured. Canopy temperature depression (CTD) was calculated using the method of Fischer et al. (1998) using the following formula:

$CTD = AT - CT$

where, *AT* and *CT* are the ambient air and canopy temperatures, respectively.

### Flag leaf greenness and estimation of stay green

A handheld leaf greenness reader (SPAD-502, from Konica Minolta Inc. of Osaka in Japan) was used to test flag leaf greenness as the Soil Plant Analysis Development (SPAD) index, from the middle portion of 10 preselected well-developed flag leaves of each plot (Fig. S3). The stay-green (SG) feature of genotypes was then derived from SPAD values of the flag-leaf using a slightly modified method of Rosyara et al. (2007)’s approach for estimating the area under the SPAD decline curve (AUSDC):

$SG = {\rm \; }\mathop \sum \limits_{i = 1}^{n - 1} \left[ {\displaystyle{{{S_{\left( i \right)}}{\rm \; } + {\rm \; }{S_{\left( {i + 1} \right)}}} \over 2}} \right]*\left[ {{D_{\left( {i + 1} \right)}} - {D_{\left( i \right)}}} \right]$

where, *S*_(*i*)_ and *S*_(*i*+1)_ are the successive flag leaf SPAD values, and *D*_(*i*)_ and *D*_(*i*+1)_ are days after sowing for *S*_(*i*)_ and *S*_(*i*+1)_ measurements, respectively.

### Canopy reflectance measurement

From 40 cm above the plant canopy, a handheld radiometer called an MS-720 by EKO Instruments, Japan, was used to measure the plant canopy’s reflectance (Fig. S3). The radiometer was used to look at the plant canopy from a 25° angle. The radiometer can collect reading from 350 to 1050 nm of the light spectrum at a 1 nm interval, and it can do this every time. The reflectance measurements were taken between 11 a.m. and 2 p.m., when there were no shadows, clouds, or strong winds. The mean of three readings from three random places in each plot was used to calculate reflectance indices. A white reflectance panel was used to figure out how much light was coming in for every canopy reflectance measurement. The calibrated reflectance values were used to make spectral reflectance indices (SRIs) under control and drought stress at five growth stages over two growing seasons. The formulae used to calculate the SRIs are shown in Table 1.

**Table 1:** Spectral reflectance indices (SRIs) assessed as indirect phenotyping for stay green, grain yield, and drought tolerance in bread wheat genotypes. SRIs were calculated under control and drought stress at heading, anthesis, 7, 14, and 21 days after anthesis (DAA) over two growing seasons.

Spectral reflectance index	Formula^a^	References
*Vegetation- & water-based SRIs*		
Simple ratio (SR)	R_800_/R_680_	Stenberg et al. (2004)
Normalized difference vegetation index (NDVI)	(R_800_ − R_680_)/(R_800_ + R_680_)	Rouse et al. (1973)
Green-NDVI (GNDVI)	(R_780_ − R_550_)/(R_780_ + R_550_)	Gitelson, Kaufman & Merzlyak (1996)
Normalized water band index (NWI)	(R_900_ − R_970_)/(R_900_ + R_970_)	Babar et al. (2006)
Plant nitrogen spectral index (PNSI)	|(R_NIR_ + R_RED_)/( R_NIR_ – R_RED_)|	Stone et al. (1996)
Enhanced vegetation index (EVI)	2.5 * (R_NIR_ − R_RED_)/(R_NIR_ + 6 * R_RED_ − 7.5 * R_BLUE_ + 1)	Liu et al. (2015)
Modified soil adjusted vegetation index (MSAVI)	0.5 * [2 * R_NIR_ + 1 − √{(2 * R_NIR_ + 1)^2^ − 8 * (R_NIR_ − R_RED_)}]	Chen (1996)
Optimized soil adjusted vegetation index (OSAVI)	(1 + 0.16) * (R_NIR_ − R_RED_)/(R_NIR_ + R_RED_ + 0.16)	Rondeaux, Steven & Baret (1996)
*Pigment specific SRIs*		
Photochemical reflectance index (PRI)	(R_530_ − R_570_)/(R_530_ + R_570_)	Peñuelas et al. (1993)
Normalized chlorophyll pigment ratio index (NCPI)	(R_680_ − R_430_)/(R_680_ + R_430_)	Peñuelas et al. (1993)
Anthocyanin reflectance index (ARI)	R_800_ * (1/R_550_ − 1/R_700_)	Gitelson, Keydan & Merzlyak (2006)
Xanthophyll pigment epoxidation state (XES)	R_531_	Penuelas, Baret & Filella (1995)
Modified carotenoid reflectance index (mCRI)	R_780_/[(1/R_510_) – (1/R_550_)]	Gitelson, Keydan & Merzlyak (2006)
Structure-insensitive pigment index (SIPI)	(R_800_ – R_445_)/(R_800_ – R_680_)	Penuelas, Baret & Filella (1995)
Plant senescence reflectance index (PSRI)	(R_678_– R_550_)/R_750_	Merzlyak et al. (1999)

**Note:**

a The letter “R” followed by three-digit numbers stands for the wavelength of respective reflectance. R_NIR_, R_RED,_ and R_BLUE_ indicate the mean reflectance of the near-infrared (770–895 nm), red (630–690 nm), and blue (450–510 nm) regions of the spectra, respectively.

### Measurement of phenotypic and yield traits

Days to heading (DTH) were calculated as the time necessary from seed sowing to the visibility of 50% of a plot’s spikes. The plant height (PH) was established by measuring the height of the completely emerging spike from base to tip, excluding the awns. At the physiological maturity, four linear meters of plants were picked at the ground level in the center of the plot. Spikes were extracted and counted from collected samples to determine the spike density per square meter of land area (NSM). Spikes were gathered in a cotton bag, and both the straw and the spikes were sun-dried. Spikes were manually cleaned after being threshed into the bag. The grain yield (GY) was calculated by weighing the grain and adjusting it to 12% moisture content. Biological yield (BY) was defined as aboveground biomass (straw + grain + chaff) that had been sun-dried, and t ha^−1^ was used to denote both BY and GY. The number of kernels per spike (NKS), the weight of kernels per spike (WKS), and the hundred kernel weight (HKW) of 10 main-shoot spikes collected separately from each plot and were determined by sun-drying, threshing, weighing, and counting grain numbers.

### Analysis of data using a general linear model and machine learning algorithms

Statistical analyses were done with META-R (multi-environment trial analysis in R) software version 6.0 (Alvarado et al., 2020) and R-4.1.0 for the win (R Core Team, 2022).The growth phase SRIs, SG, and CTD values were averaged and along with the yield traits, fitted to a combined analysis in the linear model with META-R for the randomized complete block design. The linear model used in META-R implemented in the R package lme4 (Bates et al., 2015) that uses the restricted maximum likelihood (REML) to estimate variance components (Alvarado et al., 2020). The analysis of variance (ANOVA) was carried out using the following equation to ascertain if there was a significant effect of genotype, environment (growing season, growing condition), and genotype × environment interactions on the traits:

${{\rm Y}_{ijk}} = \; \mu + \; {\rm Ge}{{\rm n}_i} + \; {\rm En}{{\rm v}_j} + {\rm Re}{{\rm p}_k}\left( {{\rm En}{{\rm v}_j}} \right) + \; ({\rm Ge}{{\rm n}_i} \times {\rm En}{{\rm v}_j}) + \; {\varepsilon _{ijk}}$

where Y_*ijk*_ is observation for the *i*-th genotype in the *j*-th environment (trial) and *k*-th replication; *µ* is the grand mean effect; Gen_*i*_ is the effect of the *i*-th genotype; Env_*j*_ is the effect of the *j*-th environment; Rep_*k*_(Env_*j*_) is the effect of the *k*-th replication within the *j*-th environment; (Gen_*i*_ × Env_*j*_) is the interaction effect of the *i*-th genotype with the *j*-th environment; and *ε*_*ijk*_ is the effect of the error associated with the *i*-th genotype, *j*-th environment and *k*-th replication, which is supposed to be independently and identically distributed (iid) normal with mean zero and variance σ^2^_*ε*_.

META-R also estimated the best linear and unbiased estimators (BLUEs) for the model simultaneously (Alvarado et al., 2020), as the estimate *(*adjusted arithmetic mean, ȳ_*ij*_) of the trait of the *i*-th genotype in *j*-th environment (Searle, 1987).

${\bar {y} _{ij.}} = \; \mu + \; {\rm Ge}{{\rm n}_i} + \; {\rm En}{{\rm v}_j} + \; ({\rm Ge}{{\rm n}_i}\; \times \; {\rm En}{{\rm v}_j})$

BLUEs were calculated for the control and drought environments across growing seasons and used in the subsequent statistical analyses. The differences between the mean BLUEs of different traits under control and drought environments were compared using the paired *t*-test and visualized in boxplots with the R packages ggplot2 and ggpubr (Wickham, 2016). The relative BLUEs (ratio of BLUE_drought_ to BLUE_control_) were used in cluster analysis, principal component analysis (PCA), and linear discriminant analysis (LDA).

The unbiased REML estimator maximizes the likelihood functions for variance components taking into account the degrees of freedom involved in calculating the fixed effects $[\sigma _{REML}^2 = \; \sum {\left( {{x_i} - \bar x} \right)^2}/\left( {n - 1} \right)]$ (Chytrasari & Kartiko, 2019). Using the REML-adjusted variance components from the above model, the broad-sense heritability across environments was calculated for SRIs and yield traits, adopting the method proposed by Alvarado et al. (2020):

${H^2} = \displaystyle{{{\rm \sigma }_g^2} \over {{\rm \sigma }_g^2 + {\rm \sigma }_{ge}^2/n{\rm Env} + {\rm \sigma }_\varepsilon ^2/\left( {n{\rm Env}\; \times n{\rm Rep}} \right)}}$

where σ^2^_*g*_, σ^2^_*ge*_, and σ^2^_*ε*_ indicate the variances of genotype, genotype × environment interaction, and error, respectively. *n*Env and *n*Rep designate number of environments and replications, respectively.

Lollipop and radar plots were created using R packages ggplot2 and fmsb, respectively, along with reshape2 (Wickham, 2016). Correlation among BLUEs of the studied parameters was visualized by the web-based MVApp application (Julkowska et al., 2019). Using the relative BLUEs of the traits (ratio of BLUE_drought_ to BLUE_control_), hierarchical clustering, principal component analysis (PCA), and linear discriminant analysis (LDA) were performed. Heatmaps and hierarchical clusters (the distance is Euclidean and the method is ward.D2) were generated by the package pheatmap (Kolde, 2019). The number of clusters was obtained prior to cluster analysis using the gap statistic algorithm in the factoextra’s fviz_nbclust function (Fig. S4). PCA was carried out using the R packages ggplot2, factoextra, and FactoMineR (Lê, Josse & Husson, 2008). LDA was performed using Minitab 19 statistical software (Minitab, 2022). ANOVA was performed among the clusters for biological and grain yields, means were compared using the estimated marginal means (EMM) test, and boxplots were constructed along with the statistics using the R packages tidyverse (Wickham et al., 2019), ggplot2-based ggpubr (Wickham, 2016), rstatix (Kassambara, 2020), and emmeans (Lenth, 2022).

## Results

### Dynamic changes in canopy reflectance

Figure S5 depicts the dynamic variations in canopy reflectance during the reproductive growth stages under drought stress and control. Both spectra have a similar typical pattern and display the usual reflectance properties of green vegetation. Canopy reflectance throughout the VIS region of the spectrum (400–700 nm) was normally low due to light absorption by leaf pigments and moderately increased by dryness, but reflectance in the NIR portion of the spectrum (700–1,050 nm) was higher and dropped dramatically under drought conditions (Fig. S5). With the progression of reproductive growth, the canopy reflectance decreased in both control and drought conditions, especially in the NIR zone of the spectra, and the decrease was substantially higher in the drought-affected plants compared to the control (Fig. S5).

### Combined analysis of variance

It was demonstrated that the genotypic main effect for all SRIs and yield parameters was extremely significant (*p* < 0.001) (Table 2). The genotype-environment interaction impact was incredibly significant (*p* < 0.001) for all SRIs, although for other yield characteristics, the interaction effect was significant at least at *p* < 0.01, with the exception of SG, PH, WKS, and HKW, for which the interaction effect was not significant (Table 2).

**Table 2:** The variance components and broad-sense heritability (H^2^) across growing seasons and conditions for SRIs, yield traits, and grain yield.

Traits	σ^2^_*g*_	σ^2^_*ge*_	σ^2^_*ε*_	σ^2^_*p*_	H^2^ (%)
SR	0.85848***	0.65401***	0.20993	1.03948	82
NDVI	0.00160***	0.00117***	0.00025	0.00191	83
GNDVI	0.00117***	0.00021***	0.00046	0.00126	92
NWI	0.00008***	0.00001***	0.00002	0.00008	94
PRI	0.00010***	0.00005***	0.00002	0.00012	87
NCPI	0.00062***	0.00046***	0.00034	0.00077	81
ARI	0.00353***	0.00440***	0.00313	0.00490	72
XES	0.39091***	0.10203***	0.23423	0.43593	89
mCRI	4571.54***	1806.83***	2359.47	5219.87	87
SIPI	0.00045***	0.00079***	0.00063	0.00070	64
PSRI	0.00009***	0.00004***	0.00009	0.00010	84
PNSI	0.00089***	0.00186***	0.00069	0.00141	63
EVI	0.00260***	0.00253***	0.00128	0.00334	77
MSAVI	0.00055***	0.00098***	0.00044	0.00083	66
OSAVI	0.00114***	0.00242***	0.00089	0.00182	62
SG	265.878***	12.0964^ns^	247.537	289.531	91
CTD	0.32023***	0.05887***	0.04501	0.33870	94
DTH	16.6434***	0.88769***	1.15631	16.9617	98
PH	52.2990***	0.00000^ns^	11.2731	53.2384	98
NSM	5307.68***	300.070**	2019.69	5551.01	95
NKS	58.9122***	6.02268***	25.9533	62.5806	94
WKS	0.17350***	0.00592^ns^	0.05317	0.17941	96
HKW	0.35090***	0.00000^ns^	0.09317	0.35867	97
BY	1.79138***	0.65522***	1.16846	2.05256	87
GY	0.75314***	0.06768**	0.50374	0.81204	92

**Note:**

**Figure 2:**
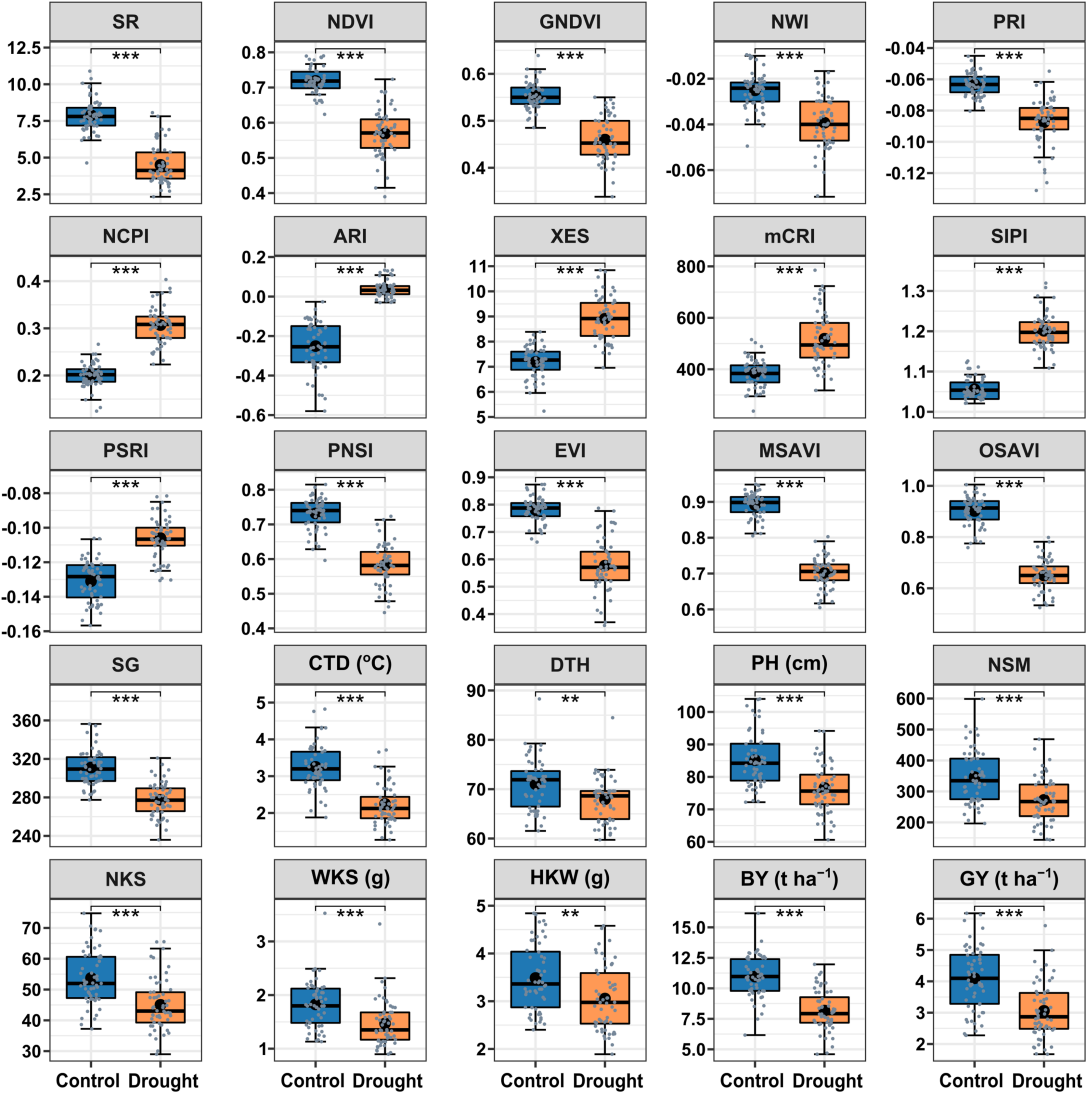
Descriptive summary of the studied spectral reflectance indices and yield traits of 56 bread wheat genotypes under control and drought treated trials in two growing seasons. Asterisks (** and ***) denote statistically significant at *p* ≤ 0.01, and *p* ≤ 0.001, respectively. Within the box, the horizontal thicker line and circle indicate the median and mean, respectively. The box’s bottom and upper boundaries, as well as the lower and upper whiskers, correspond to Q1 (first quartile/25^th^ percentile), Q3 (third quartile/75^th^ percentile), (Q1 − 1.5IQR), and (Q3 + 1.5IQR), respectively. IQR stands for interquartile range. The distribution of 56 wheat genotypes is shown by the slate color dots on the boxes. SG corresponds to stay green, CTD is for canopy temperature depression, DTH represents days to heading, PH denotes plant height in centimeters, NSM signifies spikes per square meter of land area, NKS denotes kernels per spike, WKS indicates kernel weight per spike in grams, and HKW represents hundred kernel weight in grams, BY means biological yield (t ha^–1^), and GY denotes grain yield (t ha^–1^). Table 1 has further information.

σ^2^_*g*_ = genotypic variance, σ^2^_*p*_ = phenotypic variance, σ^2^_*ge*_ = genotype × environment interaction variance, σ^2^_*ε*_ = residual/error variance. ^ns^, **, and *** Indicate non-significant, significant at *p* < 0.01 and *p* < 0.001, respectively. Additional details are shown in Table 1 and Fig. 2.

### The effect of growing environments on the SRIs and yield traits

The drought treatment showed significant differences in the best linear unbiased estimators (BLUE) of SRIs and yield traits (Fig. 2). Among the SRIs, NDVI, SR, PRI, GNDVI, NWI, PNSI, EVI, MSAVI, and OSAVI were the greatest under control and decreased critically (*p* < 0.001) due to drought stress. Conversely, NCPI, ARI, mCRI, XES, SIPI, and PSRI were the lowest in control and increased significantly (*p* < 0.001) under drought stress conditions. The SG, CTD, DTH, PH, yield attributes, BY and GY decreased significantly (*p* < 0.01) under drought conditions. Heritability in the broad sense (H^2^) was calculated over growing seasons for SRIs, yield, and yield contributing traits (Table 2), and all the traits showed a higher level of heritability (>60%) across environments, ranging from 62% to 98%. Yield traits had greater heritability (>87%), while most SRIs had a heritability of less than 90%.

### Functional relationships between mean SRIs and yield

**Figure 3:**
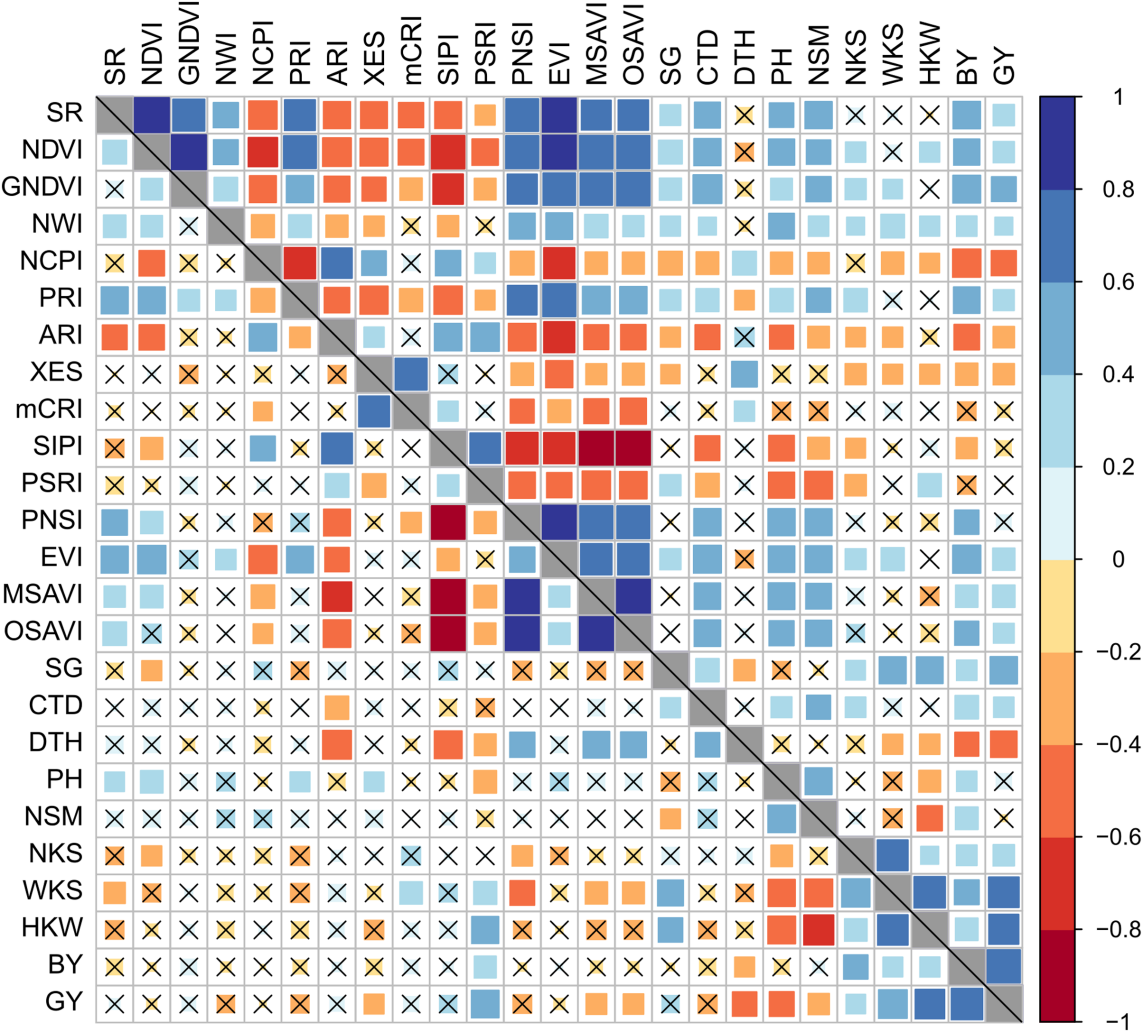
Phenotypic correlation between best linear unbiased estimators of SRIs and yield traits of 56 bread wheat genotypes under control and drought conditions. Associations under control and drought conditions are displayed in the lower and upper diagonal panels, respectively. The higher the size and color intensity of squares, the higher is the correlation coefficient. Data are averaged over two growing seasons. Crossmark (×) indicates a non-significant correlation at *p* < 0.05. Additional details are displayed in Table 1 and Fig. 2.

Both BY and GY had insignificant and irregular correlations with SRIs under control conditions; however, these correlations became significant and stronger under drought stress (Fig. 3). Among the yields, the BY exhibited a comparatively stronger association with mean SRIs under drought condition.

The BY was positively and significantly correlated (*p* < 0.05) with GNDVI, EVI, NWI, SR, NDVI, PRI, PNSI, MSAVI, OSAVI, CTD, and SG, and negatively correlated with NCPI, ARI, XES, SIPI, and PSRI under drought stress. The SR, NDVI, GNDVI, EVI, PRI, CTD, and SG were positively (*p* < 0.05) correlated with GY, while NCPI, ARI, and XES were negatively correlated under drought. The mCRI did not show any substantial correlation with either BY or GY across the growing conditions.

### Association of growth stage-specific SRIs to biological and grain yield

The growth stage-specific relationship of SRIs to BY and GY revealed that the SRIs were strongly correlated with BY and GY under drought compared to control; however, the SRIs exhibited higher correlation coefficients with BY than GY for drought treatments. Generally, SRIs taken during the mid-grain filling phases (anthesis to 14DAA) offered a stronger correlation with BY and GY related to early and late reproductive development under drought stress (Table 3). During the mid-time grain filling phases, the NWI, mCRI, SIPI, PSRI, PNSI, and MSAVI measured under drought were the SRIs having insignificant correlations with GY, whereas the insignificant relationships for BY was recorded only with mCRI, and PSRI.

**Table 3:** Correlation coefficients between SRIs and biological yield (BY) and grain yield (GY) were taken from control and drought stress treatments and measured at heading, anthesis, 7, 14, and 21 days after anthesis (DAA). The data presented are the averages of values for the two growing seasons.

SRIs		Control					Drought				
		Heading	Anthesis	7DAA	14DAA	21DAA	Heading	Anthesis	7DAA	14DAA	21DAA
SR	BY	−0.16	−0.10	−0.13	−0.13	−0.22	0.51***	0.51***	0.53***	0.38**	0.42***
	GY	−0.23	−0.14	-0.27*	−0.25	-0.29*	0.31*	0.30*	0.37**	0.35**	0.33*
NDVI	BY	0.03	0.08	−0.01	−0.03	−0.09	0.40**	0.49***	0.49***	0.45***	0.34*
	GY	−0.04	0.04	−0.06	−0.03	−0.07	0.28*	0.28*	0.39**	0.29*	0.30*
GNDVI	BY	0.22	0.25	0.13	−0.04	0.09	0.51***	0.48***	0.50***	0.43***	0.38**
	GY	0.16	0.23	0.07	−0.02	0.01	0.48***	0.41**	0.36**	0.23	0.31*
NWI	BY	−0.04	−0.12	0.05	0.07	0.09	0.14	0.17	0.31*	0.26*	0.28*
	GY	−0.17	−0.08	−0.18	−0.12	−0.08	0.03	0.04	0.29*	0.07	−0.06
NCPI	BY	−0.05	0.07	0.01	−0.05	−0.04	-0.45***	-0.46***	-0.51***	-0.47***	-0.41**
	GY	−0.05	0.22	0.04	−0.08	−0.07	-0.36**	-0.30*	-0.40**	-0.39**	−0.24
PRI	BY	−0.06	−0.01	−0.09	−0.07	−0.05	0.51***	0.50***	0.56***	0.53***	0.43***
	GY	−0.11	−0.06	−0.24	−0.25	−0.16	0.37**	0.33*	0.29*	0.19	0.18
ARI	BY	0.01	0.06	0.02	0.01	0.03	−0.25	-0.31*	-0.40**	-0.28*	−0.02
	GY	−0.07	0.10	0.14	0.11	0.11	−0.04	−0.10	-0.26*	−0.25	−0.11
XES	BY	0.02	−0.11	−0.11	−0.19	−0.16	-0.27*	-0.34*	-0.38**	-0.26*	−0.25
	GY	−0.15	-0.27*	-0.29*	−0.18	−0.18	-0.31*	-0.30*	-0.34**	-0.28*	−0.24
mCRI	BY	0.29*	0.14	0.07	0.01	−0.09	−0.20	−0.23	−0.24	−0.21	−0.14
	GY	0.32*	0.18	0.11	0.02	−0.11	−0.12	−0.11	−0.10	−0.10	0.02
SIPI	BY	0.12	0.12	0.07	0.01	0.01	−0.18	-0.26*	-0.29*	-0.27*	-0.26*
	GY	0.18	0.29*	0.23	0.15	0.09	−0.10	−0.13	−0.06	−0.09	−0.18
PSRI	BY	0.27*	0.17	0.28	0.32*	0.22	0.01	0.02	−0.19	−0.24	−0.25
	GY	0.33*	0.29*	0.47***	0.53***	0.43***	0.20	0.13	−0.02	−0.08	−0.10
PNSI	BY	−0.09	−0.12	−0.06	0.02	0.04	0.27*	0.42***	0.44***	0.36**	0.27*
	GY	-0.29*	-0.28*	−0.25	−0.14	−0.09	0.19	0.19	0.13	0.04	0.02
EVI	BY	−0.12	0.05	0.03	0.04	0.09	0.50***	0.53***	0.58***	0.56***	0.35**
	GY	−0.10	−0.10	−0.09	−0.06	−0.04	0.30*	0.34*	0.35**	0.32*	0.22
MSAVI	BY	−0.07	−0.09	−0.06	−0.05	−0.07	0.26*	0.34*	0.34*	0.48***	0.29*
	GY	−0.24	−0.25	-0.26*	−0.25	−0.24	0.12	0.15	0.08	0.05	0.03
OSAVI	BY	−0.07	0.11	−0.06	−0.04	−0.05	0.31*	0.44***	0.45***	0.37**	0.24
	GY	−0.22	−0.24	-0.29*	−0.25	−0.24	0.24	0.27*	0.20	0.08	0.01
SG	BY		0.04	−0.02	−0.08	−0.12		0.25	0.32*	0.27*	0.31*
	GY		0.13	0.25	0.15	0.11		0.41**	0.52***	0.47***	0.44***
CTD	BY	−0.22	−0.13	−0.21	−0.02	−0.07	0.30*	0.30*	0.47***	0.43***	0.38**
	GY	−0.20	−0.24	-0.33*	−0.06	−0.07	0.23	0.21	0.36**	0.37**	0.35**

**Note:**

*, **, and *** Significant at *p* ≤ 0.05, *p* ≤ 0.01, and *p* ≤ 0.001, respectively. Additional details are shown in Table 1 and Fig. 2.

### Association between yield and yield contributing traits

The association between SRIs and yield traits was determined after integrating the data as BLUEs from the two seasons. Stronger significant (*p* < 0.05) correlations among studied parameters were recorded under drought compared to the control conditions (Fig. 3). The vegetation- and water-based indices, SG, and CTD were correlated positively amongst them, whereas they correlated negatively with pigment-specific spectral indices. Both BY and GY showed significant positive correlations with vegetation- and water-based indices, SG, and CTD and negative correlations with pigment-specific indices under drought stress (Fig. 3).

BY exhibited a significant positive correlation with WKS and HKW across growing conditions, and with NKS and the number of kernels per square meter land area (NSM) under drought, whereas GY was positively correlated with WKS, HKW, and BY under both growing conditions and with NKS under drought stress (Fig. 3). Both BY and GY exhibited negative correlations with DTH under control and drought conditions. The DTH also negatively correlated with WKS and HKW, but the correlations were significant only in the drought condition (Fig. 3). A negative relationship between GY and PH was found in both growing conditions, but the association was significant only in the control condition, while PH exhibited a significant positive correlation with NSM under both growth conditions. WKS was positively correlated with NKS and HKW across the growing conditions (Fig. 3).

### Grouping of the genotypes using hierarchical cluster analysis (HCA)

**Figure 4:**
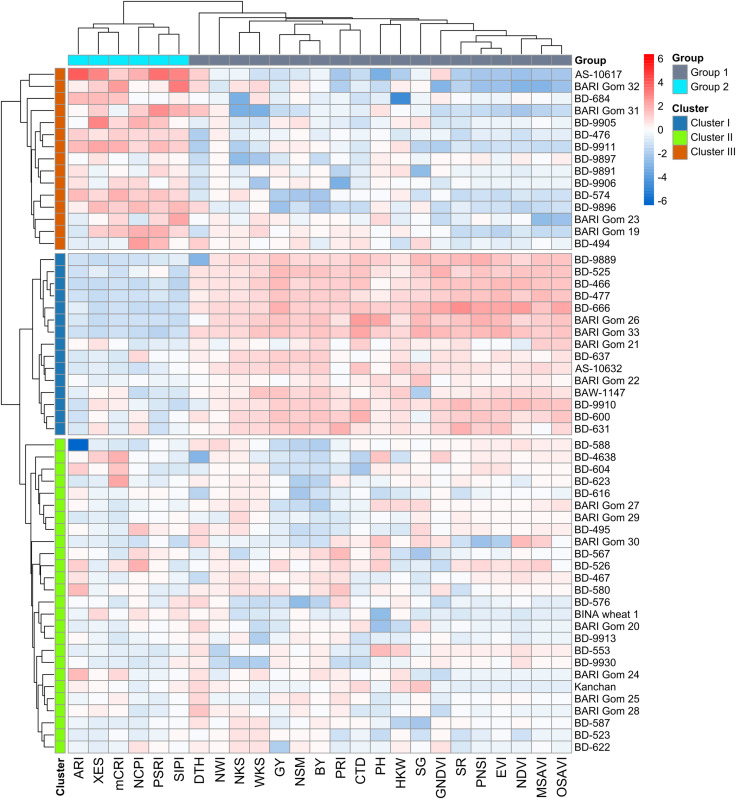
Hierarchical clustering (method = wardD2 and distance = Euclidean) and heatmap of the wheat genotypes *vs* SRIs and yield traits using relative best linear unbiased estimators. Among the clusters, genotypes of cluster I showed a greater degree of tolerance to drought followed by the genotypes of clusters II and III. Additional details are shown in Table 1 and Fig. 2.

Using the relative BLUEs of yield characteristics and SRIs, wheat genotypes were classified into separate clusters of comparable genotypes based on HCA (method = ward.D2 and distance = Euclidean) (Fig. 4). Using the machine-learning algorithm of gap statistics, the number of predicted clusters was computed (Fig. S4). Based on the dissimilarity in studied traits and SRIs, three clusters were generated at the genotype level, and two groups were separated at the variable level and presented as a two-way cluster heatmap (Fig. 4). All the yield traits and SR, NDVI, GNDVI, NWI, PRI, PNSI, EVI, MSAVI, OSAVI, SG, and CTD were placed in the variable group 1, whereas NCPI, ARI, mCRI, XES, SIPI, and PSRI confined to group 2. Cluster II has the most wheat genotypes (26) among the row clusters, followed by clusters I (15), and III (15) (Fig. 4). Generally, cluster I was determined primarily by the variables of group 1. In contrast, cluster III was determined mainly by the parameters of group 2. However, the genotypes of cluster II exhibited a diverse pattern of variations among the variables of the two studied groups (Fig. 4).

### Principal component analysis (PCA)

**Figure 5:**
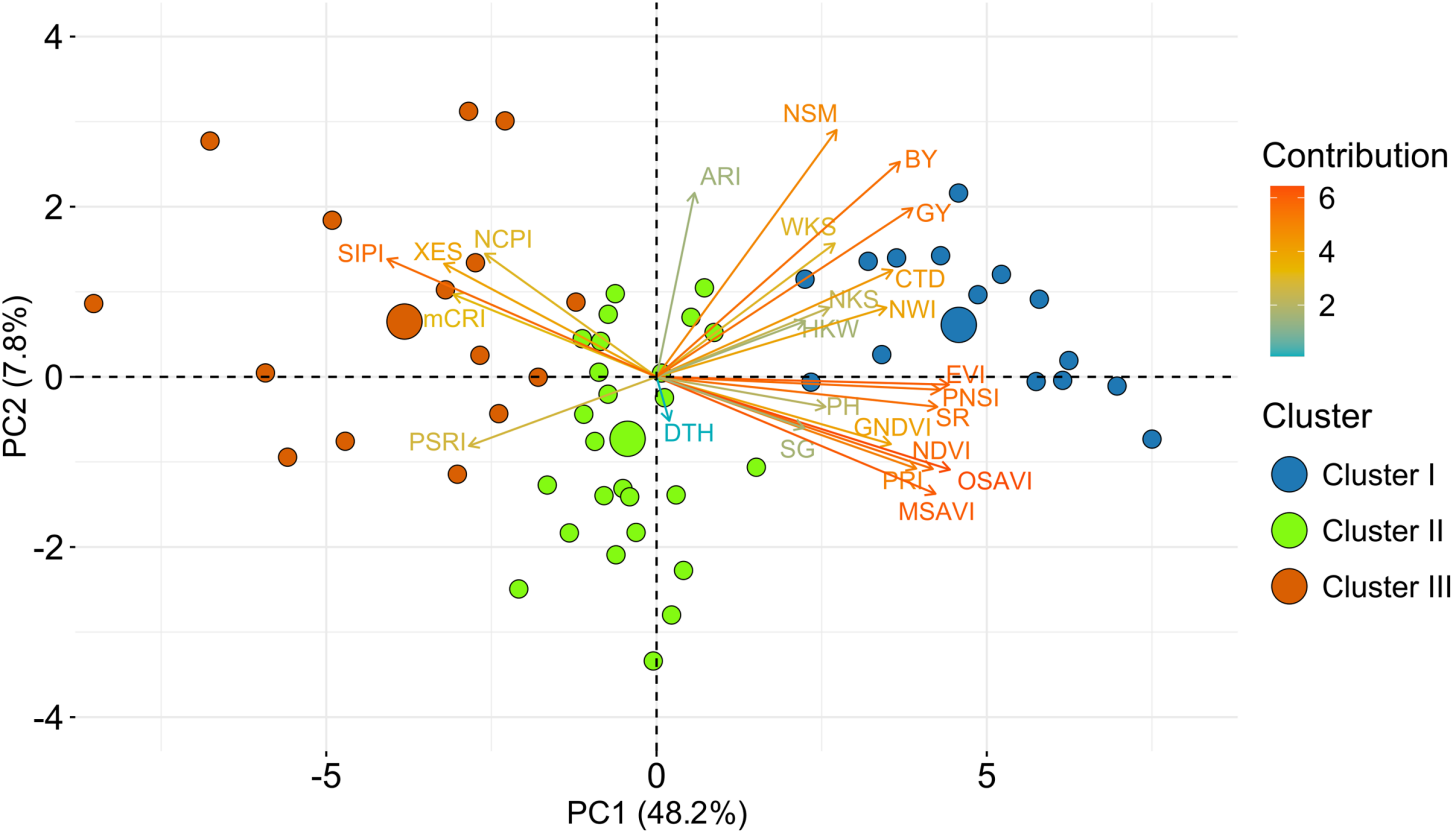
PCA-Biplot of wheat genotypes, SRIs and yield traits constructed from relative best linear unbiased estimators. Based on their dissimilarity, genotypes were distributed in distinct ordinates. In the biplot, the length indicates the quality of representation and the color intensity of a vector represents the contribution of the traits to the principal components. Positive or negative interactions of examined qualities are shown by the angles between the vectors produced from the center point of biplots. The centroid of the respective cluster is indicated by larger circles. Additional details are presented in Table 1 and Fig. 2.

The PCA based on the relative BLUE values of yield traits and SRIs unveiled a notable diversity of the bread wheat genotypes (Fig. 5). There were discovered 15 principal components (PCs), although five PCs only appeared with eigenvalues greater than one were considered significant. The first five PCs explained 73% of the variation in traits across trials, the first two of which were responsible for 56% (Table S3). In the PCA-biplot, the PC1 explained 48.2% of the total variability. All yield traits (except DTH) and SRIs contributed (positively or negatively) to the yield (excluding DTH) (Fig. 5, Table S4). The second PC, on the other hand, responded for about 7.8% variation of the total, and it was mostly contributed by ARI, NSM, BY and GY (Fig. 5, Table S3). The SRIs and yield traits that were discriminated across the first PC are assumed to be more explanatory for the evaluation of the studied genotypes.

Among the SRIs, NCPI, XES, SIPI, mCRI, and PSRI were clustered together and separated from the other attributes including BY and GY (Fig. 5), which is consistent with the negative association revealed using correlation analysis (Fig. 3). These SRIs also greatly contributed to the genotypes of cluster III (Figs. 4 and 5). Similarly, except for DTH, the positions of the rest of the traits and SRIs were placed within a 90° radius comparative to BY and GY signifying the positive phenotypic association among the traits and these traits substantially contributed to the genotypes of cluster I (Figs. 4 and 5).

### Verification of hierarchical clustering by linear discriminant analysis (LDA)

The prediction capacity of LDA was used to validate the hierarchical cluster analysis. The distribution of the genotypes within the clusters was verified and compared to identify the misclassified genotypes and to re-assigned them to suitable groups based on LDA. The LDA result indicated that genotypes were assigned to distinct clusters with 98% overall correctness (Table 4).

**Table 4:** Classification matrix of three clusters of the wheat genotypes according to linear discriminant analysis (rows are observed category and columns are predicted category).

Put into group	True group			Total no. observed
	Cluster I	Cluster II	Cluster III	
Cluster I	14	0	0	14
Cluster II	1	26	0	27
Cluster III	0	0	15	15
Total N	15	26	15	56
N correct	14	26	15	55
% correctness	93	100	100	98

The Mahalanobis squared distance (D^2^) was calculated between the clusters by LDA (Table 5). The values unveiled that clusters I and III were most distantly positioned, separated by 85.21 units, followed by clusters II and III (38.77 units). Cluster I comprised the genotypes that outperformed the majority of the phenotypes and SRIs under drought stress, while Cluster II was in the second position. On the other hand, the genotypes in cluster III appeared with poor performance (Fig. 4). The shortest distance measured in this study was between clusters I and II (36.29 units).

**Table 5:** Pairwise Mahalanobis squared distances (*D^2^*) between the clusters of wheat genotypes after dimensionality reduction by LDA.

Clusters	Cluster I	Cluster II	Cluster III
Cluster I	0	36.29^a^	85.21^a^
Cluster II	–	0	38.77^a^
Cluster III	–	–	0

**Note:**

a Distances differing from zero at a 95% confidence interval.

### Cluster-wise response in SRIs, yield traits, and grain yield to drought

**Figure 6:**
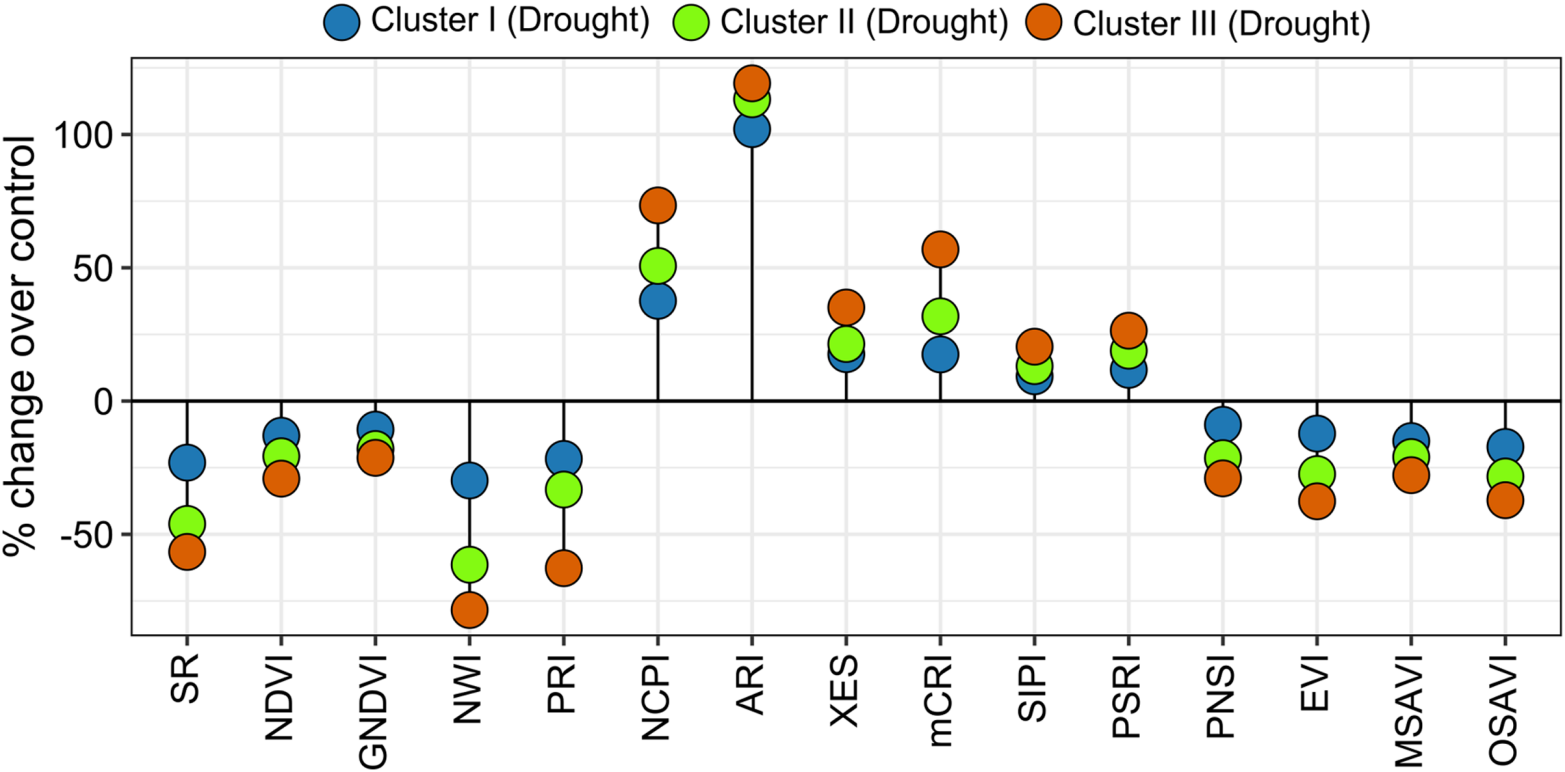
Variations in spectral reflectance indices of wheat genotypes (% change due to drought over control). Data are the means of 2 growing seasons and five growth stages for each parameter (heading, anthesis, 7, 14, and 21 DAA). Additional details are shown in Table 1.

Spectral reflectance indices which belong to group 1 in cluster analysis (SR, NDVI, GNDVI, NWI, PRI, PNSI, EVI, MSAVI, and OSAVI) declined sharply under drought, and the magnitude of decrease was lower in genotypes’ in cluster I than in clusters II and III (Figs. 4 and 6). The trend of changes over the growth stages due to drought for the above SRIs was highest in the genotypes of cluster III, intermediate in cluster II, and lowest in cluster I (Fig. S6). On the contrary, group 2 SRIs (NCPI, ARI, mCRI, XES, SIPI, and PSRI) enhanced significantly under drought-stress with a lower increment in the genotypes of cluster I compared to the genotypes of the other two clusters (Fig. 6). The increase in NCPI and XES over the growth stages was the lowest in the genotypes of cluster I, while intermediate and the highest in clusters II and III genotypes, respectively (Fig. S6).

**Figure 7:**
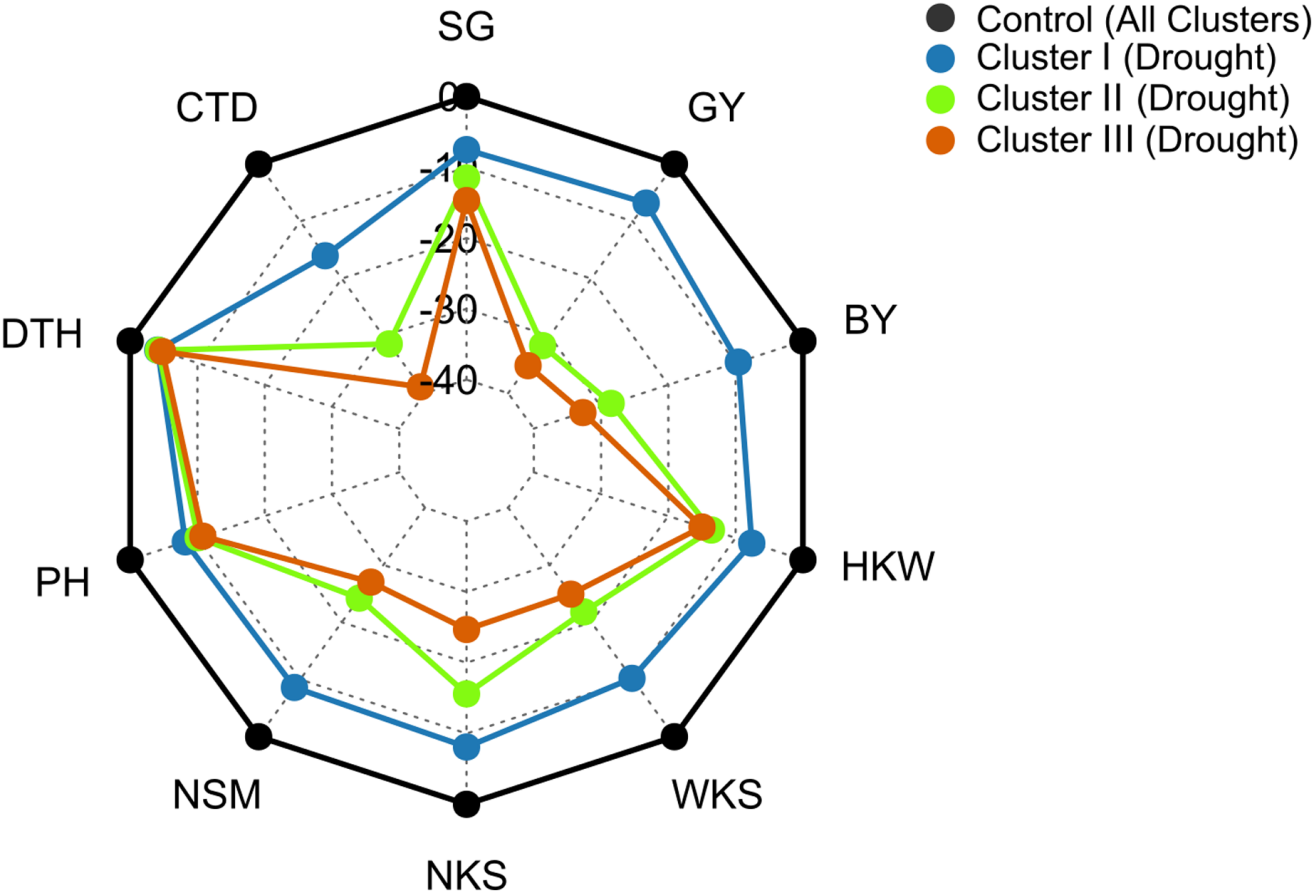
Radar plot displaying that drought stress caused differential changes in yield traits of wheat genotypes from different clusters. The values are expressed as % change over the control. Data are the best linear unbiased estimators across two growing seasons. Additional details are shown in Table 1 and Fig. 2.

Under drought conditions, DTH decreased by 4%, 4%, and 5% in clusters I, II, and III, respectively, whereas, 8%, 10%, and 11% decrease was recorded in PH (Fig. 7). The SG decreased by 8%, 13%, and 15% in the genotypes of clusters I, II, and III, respectively under drought condition (Fig. 7). Growth stage-specific change in the SG revealed comparatively a lower decline in the genotypes belonging to cluster I than the genotypes of clusters II and III under drought conditions (Fig. S6). The lowest decrease (16%) in CTD was recorded in the genotypes of cluster I, while far more decrease was observed in the genotypes of clusters II and III (31% and 39%) (Fig. 7). A consistent and lower decline in CTD was observed in cluster I compared to clusters II and III in all growth stages under drought stress (Fig. S6).

Genotypes of cluster I exhibited a comparatively lower decrease in NSM, NKS, WKS, and HKW than the genotypes of clusters II and III under drought conditions (Fig. 7). NSM decreased by 9%, 24%, and 27% in clusters I, II, and III, respectively, while 8%, 16%, and 25% decreases were recorded in NKS under drought conditions (Fig. 7). About 10% and 8% decrease in WKS and HKW, respectively, was exhibited by the genotypes of cluster I under drought conditions, whereas the decrease was 22% and 14% in cluster II, and 25% and 15% in the genotypes of cluster III (Fig. 7).

**Figure 8:**
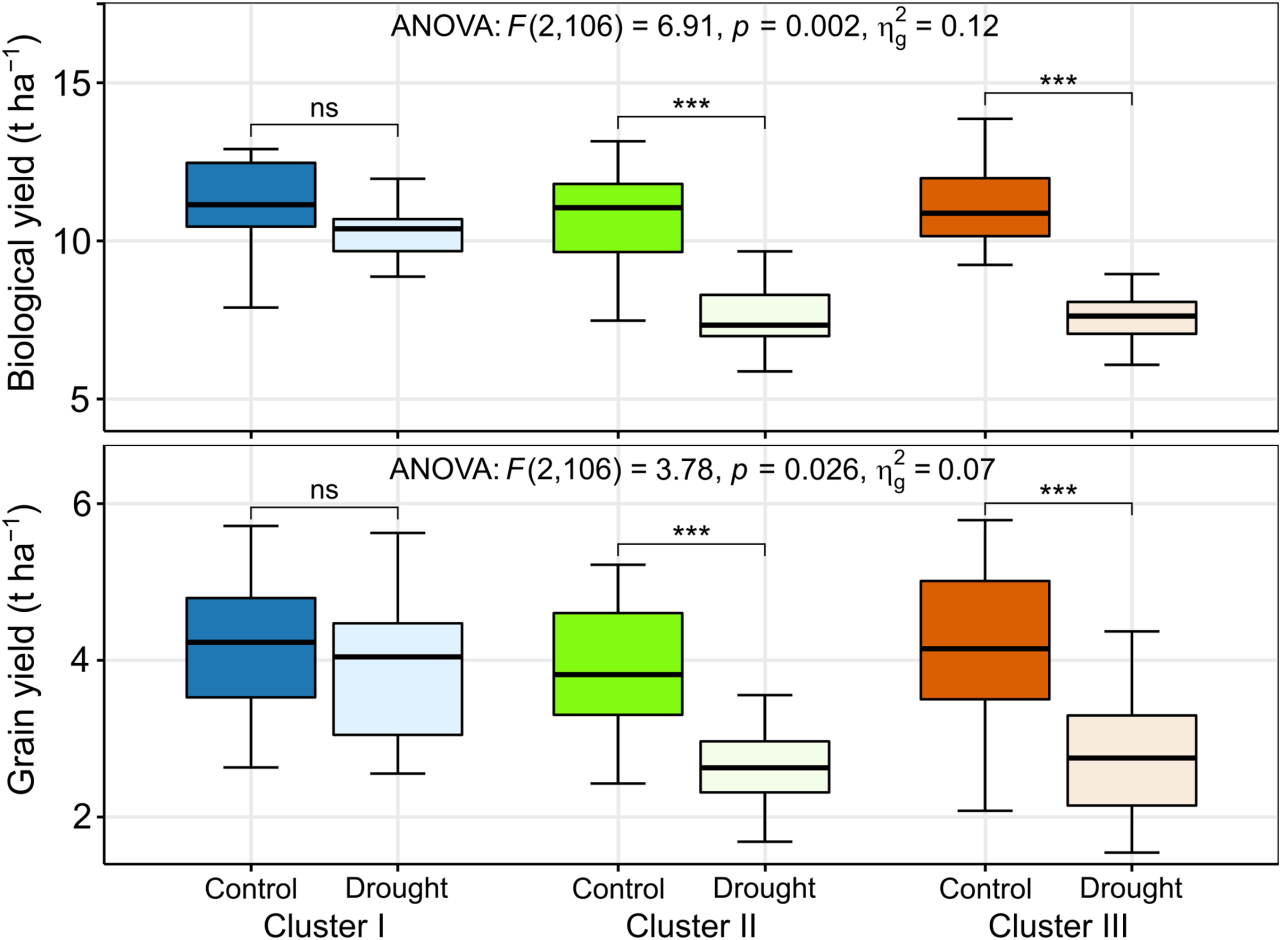
Best linear unbiased estimators of biological and grain yield of different clusters under control and drought stress. The thickened horizontal line within the box represent the median. ns and *** indicate statistically non-significant and significant at *p* ≤ 0.001, respectively, by estimated marginal means (EMM) test.

The BY decreased by 10%, 29%, and 33% in the genotypes of clusters I, II, and III, respectively, due to drought (Figs. 7 and 8). The GY for clusters I, II, and III ranged from 2.77 to 6.15 tons ha^−1^, from 2.43 to 5.22 tons ha^−1^, and from 2.08 to 5.93 tons ha^−1^ under control, and from 2.69 to 6.06 tons ha^−1^, from 1.68 to 3.95 tons ha^−1^, and from 1.55 to 4.57 tons ha^−1^ under drought, respectively (Fig. 8). The GY decreased by 7, 32, and 35% in clusters I, II, and III, respectively, due to drought (Figs. 7 and 8). The clusters and growing conditions showed significant interactions (*p* < 0.05) for BY and GY. The decreases in the BY and GY of clusters II and III were significant (*p* < 0.001) according to the EMM (estimated marginal means) test, while the decreases were not significant in the case of cluster I (Fig. 8).

## Discussion

There have been reports of the application of proximate canopy reflectance sensing to evaluate plant water status in several crops, including wheat in water deficit situations (El-Hendawy et al., 2017; Gizaw, Garland-Campbell & Carter, 2016; Gutiérrez, Reynolds & Klatt, 2010; Liu et al., 2004; Sun et al., 2019; Zhao et al., 2004; Zhou et al., 2022). Characterizing the association of SRIs with yield characters, biochemical traits, and yield of grain is important for the aggregate use of reflectance data in breeding plans for certain habitats (Gizaw, Garland-Campbell & Carter, 2016). The current study indicated that drought stress has a substantial effect on the yield characteristics and SRIs of wheat genotypes (Figs. 2 and S5), and high (>60%) broad-sense heritability (H^2^) across the years suggests less environmental effect in the observed changes of the SRIs and yield traits. Significant variations in grain yield and its attributes were also kept referring to rainfed bread wheats (Ahmed et al., 2022; Mwadzingeni et al., 2016; Prasad et al., 2007). The present study stipulated that plant vegetation and water indices were decreased under drought, however, except for PRI, a substantial increase in the pigment-specific spectral indices was recorded across growing years in our study. Gizaw, Garland-Campbell & Carter (2016) also observed the similar findings. The decrease in the plant vegetation and water indices under drought is related to the fact that these indices were computed primarily using the wavelengths of the NIR region and this region is decreased due to drought in our study (Table 1, Fig. S5). However, the increase in the pigment-specific indices is explained by their dependence mainly on the increased VIS portion of the spectrum under drought (Table 1, Fig. S5). Similar differential changes in the reflectance spectra were reported in drought-stressed wheat (El-Hendawy et al., 2017) and at various development phases of the crop (Ni et al., 2018). The higher reflectance in the VIS part of spectra in wheat under drought is due to lower pigment content and a lower leaf area index, while a lower reflectance in the NIR region relates to the changes in the leaf structure along with water absorption characteristics under drought situations (Chandel et al., 2021; El-Hendawy et al., 2017). Growth-stage specific changes in some SRIs showed that the major changes occurred at or before anthesis, and stayed more or less constant thereafter. However, the degree of change was clearly different among the tolerant groups. The distinct changes in the SRIs before anthesis implies that biomass accumulation and plant water status were affected during the onset of drought at the vegetative stage. The gradual decrease in the vegetation and water-based indices at the reproductive stages under drought reflects that the crop experienced a water-limited environment. However, an increase in the pigment-specific indices after anthesis indicated the gradual loss of leaf pigments due to drought, which can be seen in the dynamic variations in the stay green (SG) scores.

### Relation between SRIs and crop yield

Under drought, the significant phenotypic association between SRIs and grain yield and biological yield were detected across the growing years (Fig. 3). The vegetation- and water-based indices always gave positive correlation coefficients, while negative correlations occurred in the case of the pigment-specific spectral indices (NCPI, ARI, and XES). Similar relations were also reported for wheat in rainfed environments (Aparicio et al., 2000; Gizaw, Garland-Campbell & Carter, 2016; Royo et al., 2003). The indices based on vegetation and water assess the canopy’s health and water content, and the greater correlations between the SRIs with biological and grain yield suggest that canopy health and water status are important factors in a genotype’s productivity under drought conditions. Besides, the significant associations between SRIs and plant height, along with biological and grain yields also indicated that plant height and biomass development during stem elongation are the major yield determinants under limiting environment. Under poor available water, the greater correlations of vegetation- and water-based indices, CTD, and SG indicate that these indices are associated with canopy coolness and greenness. In turn, the positive correlation of CTD, SG, and grain yield imitates the cooler and greener canopies that contribute to the wheat productivity in water-limiting environments. In fact, time-resolved canopy temperature and leaf stay-green features were considered simultaneously under the short-term drought, and variations among the tested wheat genotypes were also revealed (Anderegg et al., 2021).

SRIs calculated at the mid-grain-fill stage (anthesis to 7 DAA) were, on average, strongly related to yields than SRIs measured at other growth stages (Table 3). Earlier research studies have indicated that the mid-grain-fill stage is more explanatory since genotypic differences in leaf area index, and as a result, SRIs appeared as modest in the heading and also in the early vegetative growth phases (Babar et al., 2006; Gizaw, Garland-Campbell & Carter, 2016). However, better associations between SRIs and yields were achieved when five growth stages were combined, which provided a more accurate reflection of total plant growth conditions in the growing cycle as determined by the SRIs. Nonetheless, several assessments throughout growth stages are preferable to exploit the response of numerous SRIs that collectively affect crop yield. Canopy reflectance information over different growth stages was used to forecast the yield of grains previously for wheat cultivated in spring (Babar et al., 2006) and winter wheat (Gizaw, Garland-Campbell & Carter, 2016; Sun et al., 2019).

### Relationship among CTD, SG, PH, biomass and SRIs

Canopy temperature depression (CTD) showed a significant positive correlation with all vegetation- and water-based indices (Fig. 3), imparting better plant health and water content were associated with cooler canopies resultant of higher rates of C-fixation linked with greater stomatal conductance (Bita & Gerats, 2013; Bonari et al., 2020). Apart from the SRIs, CTD also showed strong correlations with SG, biomass, plant height, yield, and yield attributes under drought in this present study. Various studies have stipulated that the CTD under drought is strongly associated with delayed leaf senescence, leaf and stem wax content, root system’s depth and spreading, the biomass of shoot, phenology, morphological attributes, spike sterility, and canopy attributes like leaf area index, plant height, coverage to ground, and orientation of leaves and infloresence, *etc*. (Lepekhov, 2022). In previous investigations, a strong association between CTD and the wheat attributes that reflect plant water status was observed (Lepekhov, 2022; Thakur, Rane & Nankar, 2022). Earlier, the water-based index NWI was suggested as an alternative to CTD by Gutiérrez-Rodríguez et al. (2004). Our findings have been reaffirmed across bread wheat genotypes, and insight on these two features combined can assist plant breeders in selecting the genotypes having higher water content and cooler canopies for water-limiting environments. SG was positively linked with vegetation- and water-based indices in the current study, except PNSI, MSAVI, and OSAVI (Fig. 3). It has been proposed that this relationship could well be utilised to create a phenotyping approach for the SG traits in wheat (Gizaw, Garland-Campbell & Carter, 2016). Staying green in wheat entails not only delayed leaf senescence but also a gradual and progressive greenness declination of the inflorescence axis and spikelets (Reynolds et al., 2009). CTD, together with canopy greenness (Kumar et al., 2017), and their temporal trends (Anderegg et al., 2021) can be exploited as a crucial feature of leaves in genotype selection for drought avoidance and tolerance to low soil moisture stress situations. Introgressing vegetation- and water-based indices, low canopy temperature, and SG traits into the wheat genotypes can produce cumulative responses that promote stress tolerance of wheat (Lopes & Reynolds, 2012), which also reflected in the results of the present investigation which revealed significantly correlated responses of the vegetation- and water-based indices with CTD and SG.

Our data also demonstrated that plant height and biomass declined substantially as a result of the drought and that they are strongly linked with the vegetation- and water-based indices. The correlated response of different vegetation indices with plant height (Ryu, Jeong & Cho, 2020) and crop biomass (Prabhakara, Hively & McCarty, 2015) under different abiotic stresses was reported in the previous studies. The significant decline in the plant height and biomass under drought could be due to stem elongation being retarded before flowering, as also indicated by the major decline in the vegetation- and water-based indices before anthesis as a result of drought stress.

### Phenotypic association of SG, CTD, and yield traits

Stay green is a key yield characteristic that permits plants to keep their leaves photosynthetically active and, as a result, improve grain filling even under stressful conditions (Borrell et al., 2014; Kamal et al., 2018; Zhang et al., 2019). In this study, SG maintained significantly positive correlations with parameters like NKS, WKS, HKW, BY, and GY (Fig. 3), implying its contribution to grain yields when water is scarced. Additionally tolerance against high temperatures and water limitence (Christopher et al., 2016; Kamal et al., 2018), the association between SG and NKS has also been reported by Luche et al. (2015). The decline in SG under drought indicates restrained photosynthetic activity, early onset of senescence, and shortened assimilate translocation to developing kernels, which ultimately contributed to yield loss. It has been observed that SG is a genotypic trait that permits agricultural plants to maintain their water status., cellular integrity, sustained photosynthetic capacity, and assimilate reallocation longer after anthesis, particularly under drought and high temperature (Christopher et al., 2008; Kamal et al., 2018). CTD is considered a trustworthy indicator of plant hydration and drought tolerance, and a positive value for CTD indicates that the cooler plant canopies than the atomosphere around (Balota et al., 2008). In the present investigation, CTD showed a positive correlation with PH, NSM, NKS, BY, and GY under drought condition (Fig. 3), suggesting the cooler canopy can be attributed to the morphological and yield traits, biomass, and grain yield during drought stress. Previous research indicated that under water limiting environment, the cooler canopies at anthesis are strongly correlated with greater spikelets spike^−1^ and a greater number of grains per spike in rain fed environment (Ozturk, 2020; Thakur, Rane & Nankar, 2022).

Drought stress generally decreases the time required to onset the heading or blooming owing to the earliler reproductive development. In this study, DTH has reduced by 3 days among the wheat genotypes due to drought. A reduced number of DTH is crucial in the breeding programs for drought stress tolerance because it allows for drought escape (Lopes & Reynolds, 2012). However, in the present study, the longer vegetative period (late heading) under control and drought did not increase grain yield, which implies that the assimilates produced over the extended pre-blooming time are not fully used during the short grain filling stage (Gizaw, Garland-Campbell & Carter, 2016; Wheeler et al., 1996). Drought-susceptible genotypes onset heading earlier during drought, resulting in a shortned life span. In contrast, drought-resistant genotypes maintained heading time which is comparable to the normal condition (Majer et al., 2008).

In the present study, drought stress negatively affected PH, NSM, NKS, WKS, HKW, BY, and GY. These results are consistent with those of Pour-Aboughadareh et al. (2020) and Wasaya et al. (2021). Reduced PH may be associated with the inhibition of cell enlargement and division, as well as faster leaf senescence, which are linked to the drought-induced reduction in turgor potential and protoplasm dehydration (Anjum et al., 2011; Khakwani et al., 2012).

In this study, significant positive correlations among NKS and WKS, HKW, BY, and GY were observed (Fig. 3), suggesting that NKS could be a reliable index of drought stress tolerance. These findings are in line with Liu et al. (2015) and Bennani et al. (2022). Earlier, NKS has been identified as the key contributor to enhanced yield in bread wheat, as well as the most affected yield traits under water-scarced environments (Chen et al., 2012; Dolferus et al., 2019). Under drought stress, reduction in the NKS along with other yield traits contributed to decreasing BY and GY (Fig. 3). The decrease in NKS is the result of floral deformities, lower viability of pollens, and fertilization inhibition induced by water scarcity throughout booting, initiation of spike, and flowering phages, which eventually accelerate yield loss (Dolferus, Ji & Richards, 2011; Dolferus et al., 2019).

### Classification and selection of genotypes

Instead of looking at each trait separately, we adopted multivariate techniques to look at all of them together. Cluster analysis classifies genotypes that are similar in a cluster and the extracted clusters are distinct from each other. In the present study, the hierarchical cluster analysis yielded three different clusters from 56 wheat genotypes by evaluating the contribution of the studied traits (Fig. 4). Cluster I has the best ability to withstand drought, as determined by the lower magnitude of changes in SRIs and yield traits, and hence is designated as drought-tolerant. In contrast, clusters II and III are the moderately tolerant and susceptible clursters, respectively (Figs. 5 and 6). Cluster analysis was used to establish genetic divergence and classification of drought tolerance in wheat by Grzesiak et al. (2019), Islam et al. (2021) and Mohi-Ud-Din et al. (2021). The subsequent linear discriminant analysis exhibited that genotypes were assigned to different clusters with 98% correctness, which verified there was no significant misclassification error while clustering the genotypes (Table 4). Mahalanobis distance matrix (D^2^) unveiled that the clusters were significantly different from one another (Table 5).

The PCA-biplots clearly visualized the relationship between the SRIs and yield traits, as well as the coordinates of the wheat genotypes in the components (Fig. 5). All of the vegetation and water-based indices were within a 90° radius of BY and GY, indicating a positive phenotypic association. Because of their physiological relationship to vegetative growth, biomass, water content, and grain yield, vegetation and water-based indices, yield attributes, and yields were clustered into one dimension (Fig. 5). The close proximity among the vegetation and water-based indices indicates strong co-linearity and potential information redundancy. Similar vicinity was also found in the case of pigment-specific indices. This analysis may aid in the development of a dimension-reduced prediction model for grain yield under drought conditions. However, further evaluation is warranted to decrease dimensionality and information redundancy by defining unique indices that reflect different physiological features. According to the previous research, many of these indices are linked to various physiological aspects of yields and yield stability, such as NDVI to biomass, pigments, and SG; NCPI to photosynthetic efficacy under stress; NWI to crop water status (Babar et al., 2006; Reynolds, Pask & Mullan, 2012).

Thus, it is evident that 56 genotypes of wheat showed substantial differencein the SRIs and yield traits under drought and the multivariate analyses effectively classified the wheat genotypes into three clusters differing in drought tolerance. Among the genotypes, those of cluster I exhibited minimal changes in SRIs and yield traits, imparting better yield stability under drought compared to the genotypes of the other two clusters (Figs. 4 and 8), which perpetuated the relative tolerance of the genotypes of cluster I to drought.

## Conclusions

To understand the mechanisms of tolerance to low water potential, the study underlined the importance of the phenotypic association of SRIs with yield traits. Under drought conditions, positive associations were observed among grain yield, vegetation- and water-based SRIs, SG, and CTD, making these traits potential physiological indicators that can be employed to boost yield capacity and tolerance to drought of bread wheat. Based on multivariate analyses, cluster I genotypes appeared to be drought-tolerant when compared to clusters II and III genotypes, because these genotypes showed a lower degree of change in SRIs and yield traits in response to drought. The findings of this study could help to design an indirect selection approach for drought tolerance and yield stability based on the association between SRIs and grain yield.

## Supplemental Information

10.7717/peerj.14421/supp-1 Supplemental Information 1 Raw data.

10.7717/peerj.14421/supp-2 Supplemental Information 2 List of genotypes and ANOVA table.

## Additional Information and Declarations

### Data Availability

The following information was supplied regarding data availability: The raw measurements are available in the Supplemental Files.
