# Supplementary material for: Canopy spectral reflectance indices correlate with yield traits variability in bread wheat genotypes under drought stress
Source: PeerJ. 2022 Nov 25;10:e14421. doi: 10.7717/peerj.14421 (PMC9703988; doi:10.7717/peerj.14421)
Supplement: Supplemental Information 2 [file peerj-10-14421-s002.pdf]

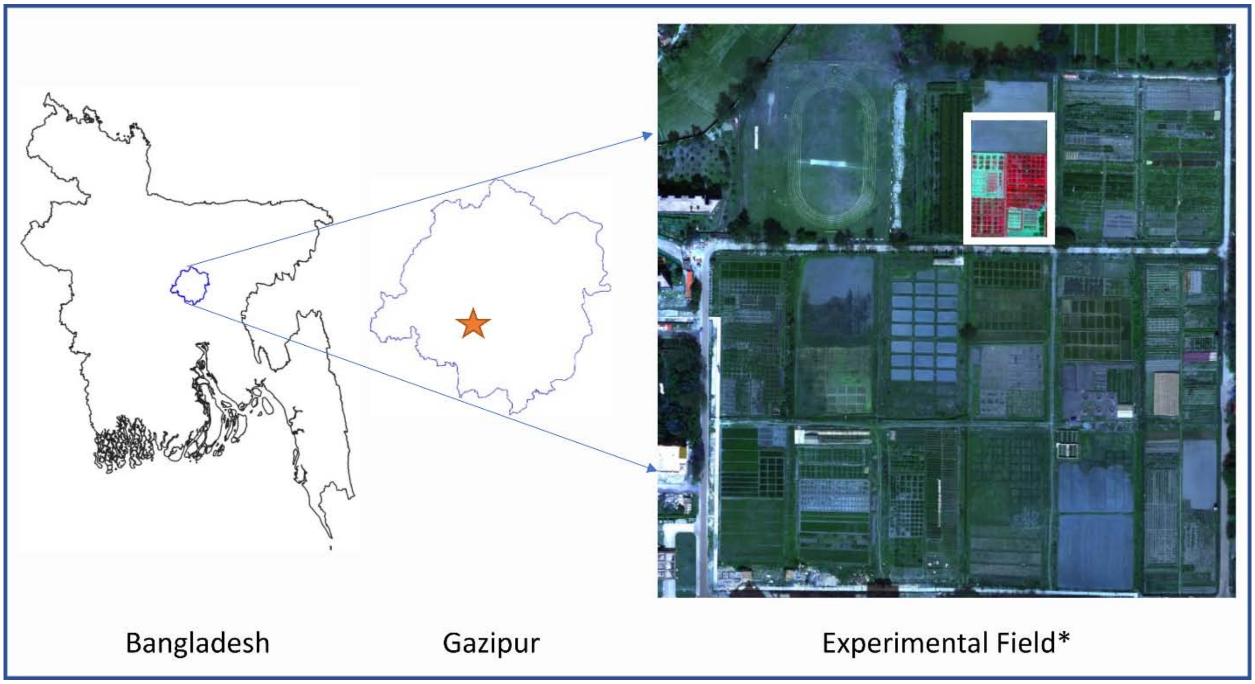

Figure S1: Location map showing the field laboratory of the Department of Crop Botany (white rectangle), Bangabandhu Sheikh Mujibur Rahman Agricultural University (24.038 °N latitude, 90.397 °E longitudes), Gazipur, Bangladesh. * the aerial photograph is taken by the author using an unmanned aerial vehicle (UAV) in March 2022 and does not represent the experimental layout of the present study.

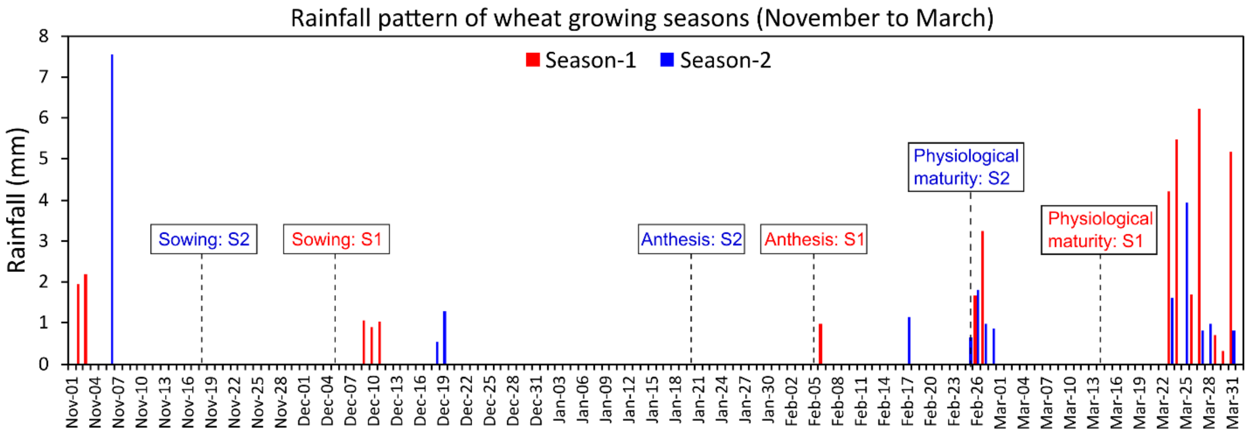

Figure S2: Daily rainfall patterns of the experimental site in two wheat growing seasons (1 November to 31 March). S1 and S2 represent wheat growing season 1 and 2, respectively.

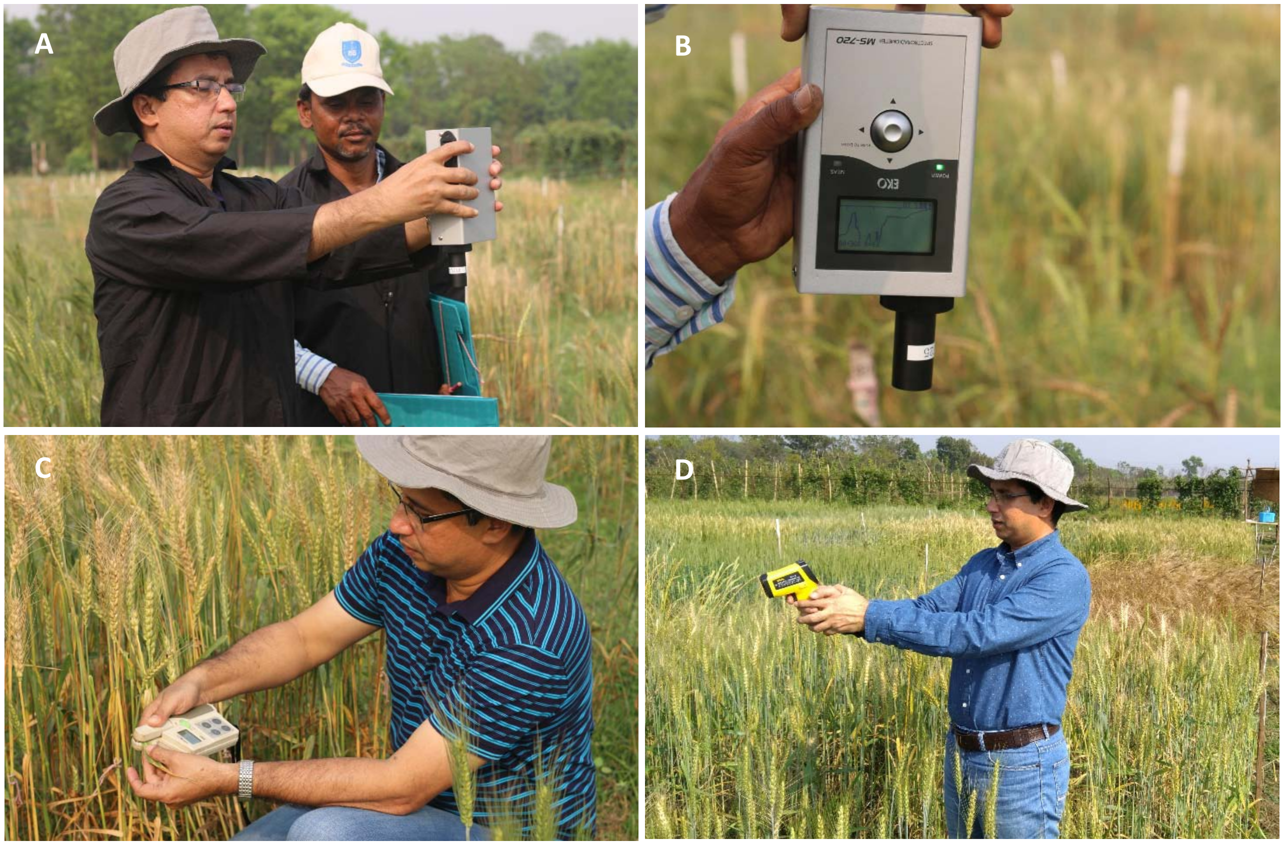

Figure S3: Field measurement of canopy reflectance using a spectroradiometer (A, B); SPAD chlorophyll index (C); and canopy temperature (D).

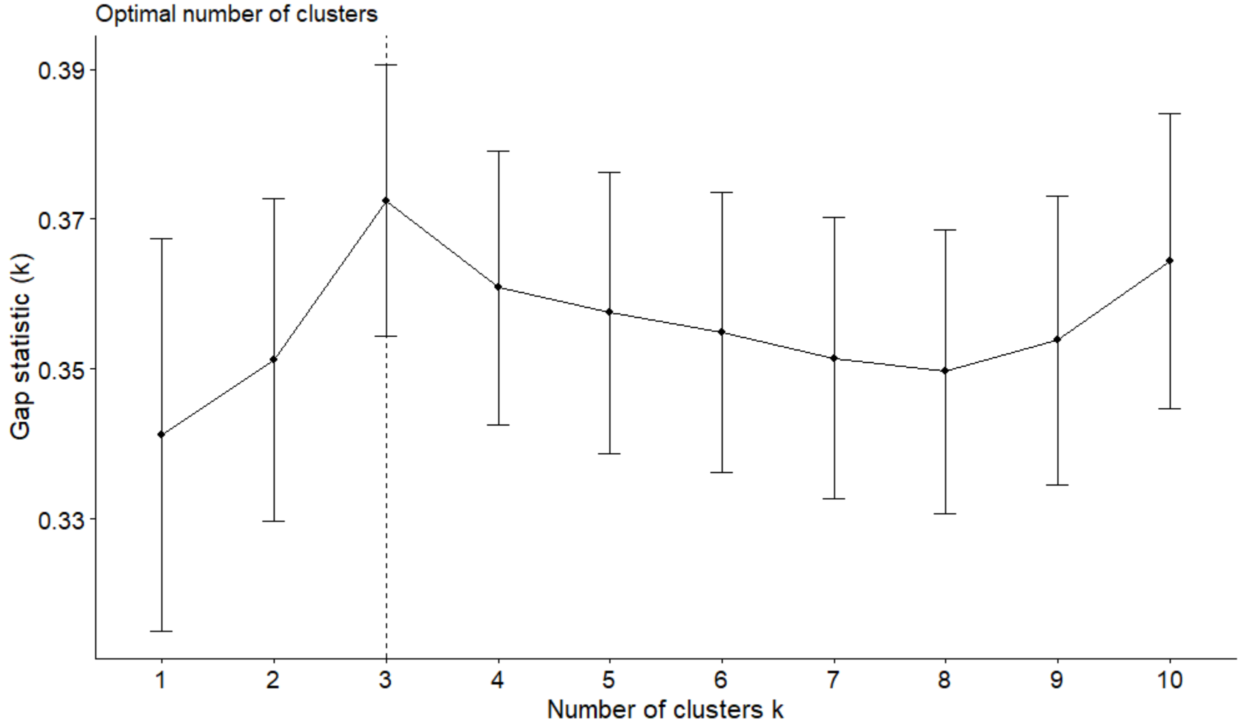

Figure S4: Gap statistic showing an optimal number of clusters to be created in the hierarchical cluster analysis based on the relative best linear unbiased estimators(BLUE)values of the SRIs and yield traits.

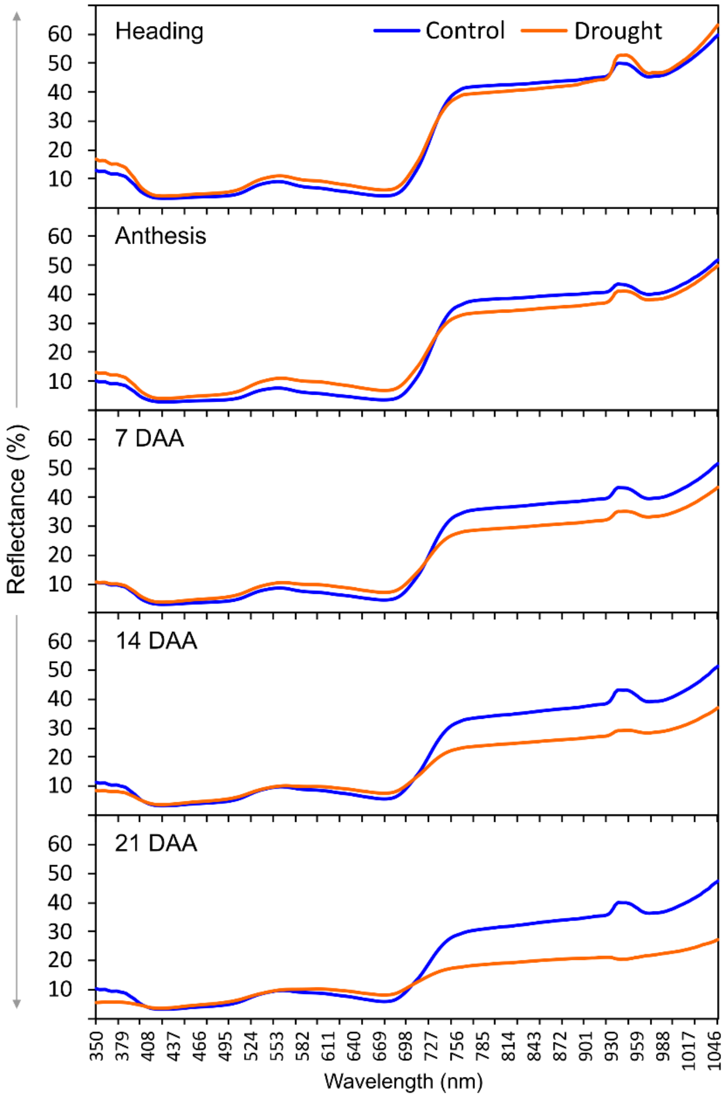

Figure S5. Dynamic changes in the reflectance spectra for wheat genotypes subjected to control and drought stress at the reproductive growth stages viz. heading, anthesis, 7 days after anthesis (DAA), 14DAA, and 21DAA. The data are averaged over two growing years. DAA denotes days after anthesis.

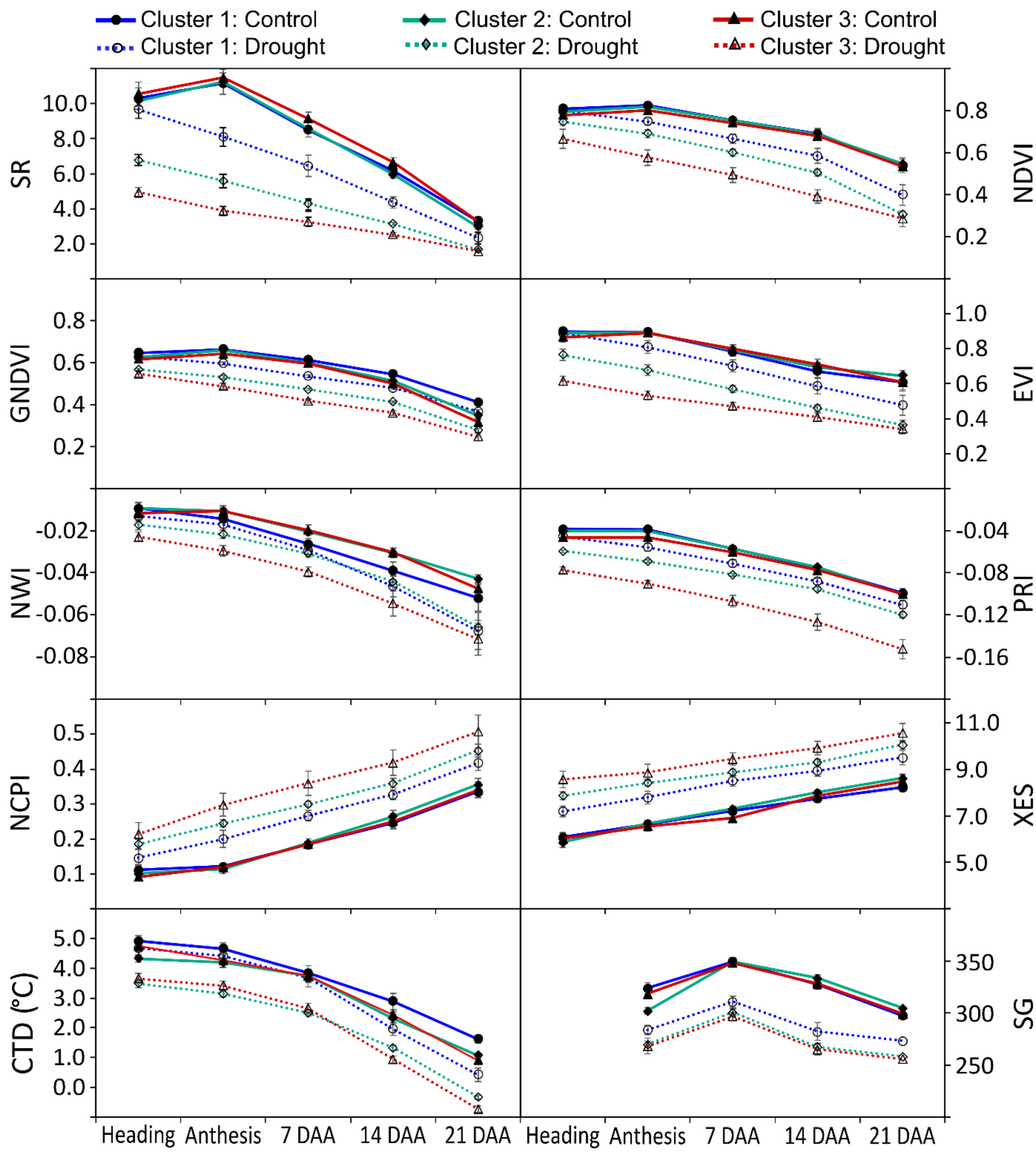

Figure S6. The growth stage-specific changes in the SRIs of different clusters of 56 wheat genotypes measured under control and drought stress. SRIs were estimated as the best linear unbiased estimatorsacross two growing seasons. Additional details are shown in Table 1 and Figure 2.

Table S1: List of wheat genotypes used in the exploratory study

Sl. No.	Genotype	Source	Type	Pedigree/Collected from
1	AS-10617	ACI Seed	Mutant	BARIGom 25; mutagen: 1% EMS
2	AS-10632	ACI Seed	Mutant	BARIGom 25; mutagen: 1% EMS
3	BARI Gom 19	BWMRI	Variety	NAC/VEE (NL 560)
4	BARI Gom 20	BWMRI	Variety	TURACO/CHIL
5	BARI Gom 21	BWMRI	Variety	MRNG/BVC//BLO/PVN/3/PJB-81
6	BARI Gom 22	BWMRI	Variety	KAN/6/COQ/F61.70//CNDR/3/OLN/4/ PHO/5/MRNG/ALDAN//CNO
7	BARI Gom 23	BWMRI	Variety	NL297*2/LR25
8	BARI Gom 24	BWMRI	Variety	G. 162/BL 1316//NL 297
9	BARI Gom 25	BWMRI	Variety	ZSH 12/HLB 19//2*NL297
10	BARI Gom 26	BWMRI	Variety	ICTAL 123/3/RAWAL 87//VEE/HD2285
11	BARI Gom 27	BWMRI	Variety	WAXWING*2/VIVISTI
12	BARI Gom 28	BWMRI	Variety	CHIL/2*STAR/4/BOW/CROW//BUC/P VN/3/2*VEE#10
13	BARI Gom 29	BWMRI	Variety	SOURAV/7/KLAT/SOREN//PSN/3/BO W/4/VEE#5. 10/5/CNO 67/MFD// MON/3/ SERI/6/NL297
14	BARI Gom 30	BWMRI	Variety	BAW 677/Bijoy
15	BARI Gom 31	BWMRI	Variety	KAL/BB/YD/3/PASTOR
16	BARI Gom 32	BWMRI	Variety	SHATABDI/GOURAB
17	BARI Gom 33	BWMRI	Variety	KACHU/SOLALA
18	BAW-1147	BWMRI	Advanced line	WRS, Gazipur
19	BD-4638	PGRC, BARI	Accession	-
20	BD-466	PGRC, BARI	Accession	-
21	BD-467	PGRC, BARI	Accession	-
22	BD-476	PGRC, BARI	Accession	-
23	BD-477	PGRC, BARI	Accession	-
24	BD-494	PGRC, BARI	Accession	-
25	BD-495	PGRC, BARI	Accession	-
26	BD-523	PGRC, BARI	Accession	-
27	BD-525	PGRC, BARI	Accession	-
28	BD-526	PGRC, BARI	Accession	-
29	BD-553	PGRC, BARI	Accession	-

Sl. No.	Genotype	Source	Type	Pedigree/Collected from
30	BD-567	PGRC, BARI	Accession	-
31	BD-574	PGRC, BARI	Accession	-
32	BD-576	PGRC, BARI	Accession	-
33	BD-580	PGRC, BARI	Accession	-
34	BD-587	PGRC, BARI	Accession	-
35	BD-588	PGRC, BARI	Accession	-
36	BD-600	PGRC, BARI	Accession	-
37	BD-604	PGRC, BARI	Accession	-
38	BD-616	PGRC, BARI	Accession	-
39	BD-622	PGRC, BARI	Accession	-
40	BD-623	PGRC, BARI	Accession	-
41	BD-631	PGRC, BARI	Accession	-
42	BD-637	PGRC, BARI	Accession	-
43	BD-666	PGRC, BARI	Accession	-
44	BD-684	PGRC, BARI	Accession	-
45	BD-9889	PGRC, BARI	Accession	RARS, Ishurdi
46	BD-9891	PGRC, BARI	Accession	RARS, Ishurdi
47	BD-9896	PGRC, BARI	Accession	RARS, Ishurdi
48	BD-9897	PGRC, BARI	Accession	RARS, Ishurdi
49	BD-9905	PGRC, BARI	Accession	RARS, Ishurdi
50	BD-9906	PGRC, BARI	Accession	RARS, Ishurdi
51	BD-9910	PGRC, BARI	Accession	RARS, Ishurdi
52	BD-9911	PGRC, BARI	Accession	RARS, Ishurdi
53	BD-9913	PGRC, BARI	Accession	RARS, Ishurdi
54	BD-9930	PGRC, BARI	Accession	RARS, Ishurdi
55	BINA wheat 1	BINA	Variety	L-880-43
56	Kanchan	BWMRI	Variety	UP301/C306

ACI- Advanced Chemical Industries; BARI- Bangladesh Agricultural Research Institute; BINA- Bangladesh Institute of Nuclear Agriculture; BWMRI- Bangladesh Wheat and Maize Research Institute; PGRC- Plant Genetic Resource Center; RARS- Regional Agricultural Research Station; WRS- Wheat Research Station.

Table S2: Monthly average climatic data across two wheat-growing seasons andthe averages of tensuccessive years (2010 – 2019)in the experimental site (24.038°N, 90.397°E).

Month	Air Temperature (°C)			Soil Temp. (°C) (30 cm depth)	Humidity (%)	Total Rainfall (mm)	Daily mean evaporation (mm)
	Max	Min	Mean				
Average across two growing seasons							
November	29.4	17.7	23.6	23.9	88.3	11.7	2.4
December	25.8	14.2	20.0	19.8	88.3	4.9	1.6
January	24.9	11.1	18.0	16.9	86.7	0.0	1.7
February	28.2	15.1	21.7	19.2	84.9	7.5	2.7
March	31.9	19.3	25.6	23.2	84.0	19.6	3.9
The ten-year average							
November	29.0	18.9	23.9	24.9	84.3	15.9	2.2
December	25.0	13.8	19.4	20.9	86.6	8.3	1.5
January	22.9	11.4	17.2	18.2	87.2	3.2	1.6
February	27.7	15.6	21.6	20.5	83.0	11.0	2.8
March	31.7	20.9	26.3	23.7	80.2	50.2	3.8

Source: BSMRAU Weather Station, Department of Agricultural Engineering, BSMRAU.

Table S3: Extracted Eigenvalues and latent vectors of the PCA conducted using the relative values of best linear unbiased estimators (BLUE) of spectral reflectance indices and yield traits associated with the first five principal components

Variable	Principal components				
	PC1	PC2	PC3	PC4	PC5
Extracted Eigenvalues	12.05	1.95	1.63	1.34	1.17
Explained variance (%)	48.2	7.8	6.5	5.4	4.7
Cumulative variance (%)	48.2	56.0	62.5	67.9	72.6
Traits and indices	*Latent vectors*				
SR	0.882	-0.073	0.063	-0.138	0.058
NDVI	0.868	-0.223	0.193	-0.086	-0.102
GNDVI	0.736	-0.164	0.075	-0.093	0.056
NWI	0.722	0.169	-0.226	0.072	-0.160
PRI	0.816	-0.224	-0.086	0.034	0.256
NCPI	-0.593	0.266	0.081	-0.064	-0.169
ARI	0.119	0.449	0.379	0.316	-0.562
mCRI	-0.638	0.201	0.342	0.170	0.386
XES	-0.704	0.250	0.202	-0.191	0.048
SIPI	-0.846	0.287	-0.151	0.015	-0.026
PSRI	-0.589	-0.168	-0.014	-0.077	0.383
PNSI	0.891	-0.031	0.225	-0.075	-0.006
EVI	0.918	-0.019	0.169	-0.049	0.088
MSAVI	0.876	-0.285	0.133	-0.118	0.010
OSAVI	0.921	-0.226	0.097	-0.125	0.047
SG	0.464	-0.125	-0.273	0.197	-0.278
CTD	0.741	0.260	-0.293	0.073	-0.163
DTH	0.040	-0.107	-0.841	-0.063	-0.114
PH	0.531	-0.073	0.394	0.173	-0.206
NSM	0.566	0.602	-0.093	-0.402	0.132
NKS	0.543	0.169	-0.165	0.597	0.295
WKS	0.560	0.326	0.007	0.439	0.344
HKW	0.466	0.136	-0.102	0.385	0.077
BY	0.651	0.598	-0.127	-0.311	0.106
GY	0.767	0.440	0.072	-0.224	0.077
